# Two antagonistic response regulators control *Pseudomonas aeruginosa* polarization during mechanotaxis

**DOI:** 10.15252/embj.2022112165

**Published:** 2023-02-16

**Authors:** Marco J Kühn, Henriette Macmillan, Lorenzo Talà, Yuki Inclan, Ramiro Patino, Xavier Pierrat, Zainebe Al‐Mayyah, Joanne N Engel, Alexandre Persat

**Affiliations:** ^1^ Institute of Bioengineering and Global Health Institute, School of Life Sciences, Ecole Polytechnique Fédérale de Lausanne Lausanne Switzerland; ^2^ Department of Medicine University of California San Francisco CA USA; ^3^ Department of Microbiology and Immunology University of California San Francisco CA USA

**Keywords:** cell polarity, mechanosensing, response regulators, twitching motility, type IV pili, Cell Adhesion, Polarity & Cytoskeleton, Microbiology, Virology & Host Pathogen Interaction, Signal Transduction

## Abstract

The opportunistic pathogen *Pseudomonas aeruginosa* adapts to solid surfaces to enhance virulence and infect its host. Type IV pili (T4P), long and thin filaments that power surface‐specific twitching motility, allow single cells to sense surfaces and control their direction of movement. T4P distribution is polarized to the sensing pole by the chemotaxis‐like Chp system via a local positive feedback loop. However, how the initial spatially resolved mechanical signal is translated into T4P polarity is incompletely understood. Here, we demonstrate that the two Chp response regulators PilG and PilH enable dynamic cell polarization by antagonistically regulating T4P extension. By precisely quantifying the localization of fluorescent protein fusions, we show that phosphorylation of PilG by the histidine kinase ChpA controls PilG polarization. Although PilH is not strictly required for twitching reversals, it becomes activated upon phosphorylation and breaks the local positive feedback mechanism established by PilG, allowing forward‐twitching cells to reverse. Chp thus uses a main output response regulator, PilG, to resolve mechanical signals in space and employs a second regulator, PilH, to break and respond when the signal changes. By identifying the molecular functions of two response regulators that dynamically control cell polarization, our work provides a rationale for the diversity of architectures often found in non‐canonical chemotaxis systems.

## Graphical abstract

The chemotaxis‐like sensing system Chp localizes and activates the type IV pili extension regulators PilG and PilH to determine the direction of *Pseudomonas* motility.

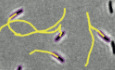

## Introduction

Bacteria use mechanosensing to rapidly adapt to life on surfaces (Persat *et al*, 2015b; Dufrêne & Persat, 2020). Mechanosensory systems regulate virulence, adhesion, biofilm formation, and surface‐specific motility (Wolfgang *et al*, 2003; Siryaporn *et al*, 2014; O'Toole & Wong, 2016; Laventie *et al*, 2019). The opportunistic pathogen *Pseudomonas aeruginosa* senses surfaces using extracellular filaments called type IV pili (T4P; Persat *et al*, 2015a; Koch *et al*, 2022). *P. aeruginosa* responds to T4P‐mediated mechanosensing on two timescales. Mechanosensing controls the direction of surface‐specific twitching motility within seconds in a process called mechanotaxis (Kühn *et al*, 2021). It also regulates the transcription of a series of genes responsible for acute virulence and surface adaptation through the production of the second messenger cyclic adenosine monophosphate (cAMP) over minutes to hours (Wolfgang *et al*, 2003; Bertrand *et al*, 2010; Fulcher *et al*, 2010; Persat *et al*, 2015a).

To power surface‐specific twitching motility, T4P successively extend, attach, and retract, thereby pulling a cell forward (Merz *et al*, 2000; Burrows, 2012). During twitching, individual *P. aeruginosa* cells can move forward persistently and reverse spontaneously or upon collision (Kühn *et al*, 2021). To twitch forward, *P. aeruginosa* localizes the T4P extension motor PilB to the cell pole facing the direction of motion. We refer to this asymmetric protein and T4P localization as polarization.

During mechanotaxis, T4P generate mechanical signal input, possibly upon surface attachment or during extension/retraction. A chemotaxis‐like system called Chp relays T4P signals to cellular components that ultimately control twitching and cAMP levels (Darzins, 1994; Whitchurch *et al*, 2004). One output response is the deployment of additional T4P, which thereby establishes a positive feedback loop. In response to signal input at one pole, Chp controls the distribution of T4P extension proteins to assemble T4P at that same pole. As a result of this polarized T4P deployment, single cells twitch persistently forward (Kühn *et al*, 2021). To change twitching direction spontaneously or upon collision, cells reverse polarization (Movie EV1). To enforce the connection between T4P input and T4P polarization output, Chp employs two response regulators PilG and PilH that establish a local excitation – global inhibition signaling landscape (Kühn *et al*, 2021). This signaling architecture is shared with higher‐order organisms: neutrophils, amoebae, and yeast polarize via local excitation global inhibition during chemotaxis or cell division (Levchenko & Iglesias, 2002).

While the sensory system controlling flagellar rotation in *Escherichia coli*, the Che system, serves as a model chemotaxis system to understand more complex ones, it is more of an exception than the norm. Bacteria possess a wide diversity of chemotaxis systems with different inputs, outputs, and overarching signaling mechanisms. Our knowledge of the dynamic control of these diverse chemotaxis‐like systems is however limited. Chp shows important functional and structural differences with the canonical Che sensory system despite strong homology (Bi & Sourjik, 2018). First, Chp responds to a mechanical signal input from T4P. Second, the Chp complex has more components than Che. CheA, the histidine kinase of the Che system, phosphorylates a single response regulator to relay input signals to its functional target. The histidine kinase ChpA comprises six phosphotransfer domains against just one for CheA (Whitchurch *et al*, 2004). In addition, Chp possesses not one but two response regulators, PilG and PilH, with opposing functions in mechanotaxis and cAMP‐dependent transcription (Darzins, 1994; Bertrand *et al*, 2010; Fulcher *et al*, 2010; Kühn *et al*, 2021). PilG promotes persistent forward twitching and cAMP production, while PilH promotes reversals and reduces cAMP levels. However, how ChpA activates PilG and PilH to control cell polarization upon mechanosensing remains unresolved.

To guide twitching direction, T4P bind to the substrate and activate the Chp system, possibly through the interaction of the major pilin PilA and the Chp system receptor PilJ (Persat *et al*, 2015a). As a result, PilJ stimulates ChpA autophosphorylation. ChpA then activates PilG and PilH presumably by direct interaction and phosphotransfer (Silversmith *et al*, 2016). PilG and PilH activate their targets, which ultimately mediate polarization of the extension motor PilB and its activator FimX (Kühn *et al*, 2021), although direct interactions have yet to be rigorously identified (Jain *et al*, 2017). PilG promotes recruitment of both PilB and FimX at the pole, while PilH inhibits it (Kühn *et al*, 2021). As a consequence, T4P extend preferentially at that same pole, resulting in persistent forward twitching. Without a counterpart to PilG, i.e., in *pilH* deletion mutants, cells continue twitching in one direction without reversing (Kühn *et al*, 2021).

Despite homologies and analogies with other well‐studied chemotaxis systems, how antagonistic response regulators establish a stable yet dynamic signaling landscape remains unknown. In particular, how PilG and PilH help transduce mechanical signal input into cell polarization output that dynamically controls the direction of *P. aeruginosa* twitching is unresolved. Here, we investigate how phosphorylation by ChpA activates PilG and PilH to regulate cell polarity in response to mechanical signal input. We combined bacterial genetics with microscopy to determine the subcellular localizations and polarization of the two response regulators in their different active and inactive states. We suggest a model in which one regulator acts as primary signal relay, while the other regulator only modulates the function of the first one.

## Results

### PilG and PilH localization during surface adaptation

**Figure 1:**
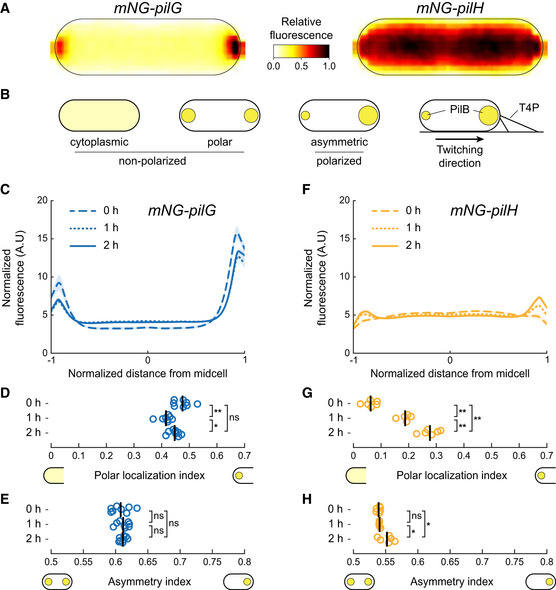
PilG and PilH localization during surface adaptation A
Average normalized fluorescent signal over 42 cells (bright pole to the right) for mNG‐PilG and mNG‐PilH transferred from liquid culture to a solid substrate and imaged immediately.B
Schematic classification of localization patterns. The spot size indicates the relative protein concentration.C–H
(C, F) Normalized average fluorescence profiles for mNG‐PilG and mNG‐PilH of hundreds to thousands of cells (cf. Appendix Table S4). The length of each cell is normalized so that the dim pole is positioned at *x* = −1, the bright pole at *x* = 1. The fluorescent profile for each cell is normalized to its total fluorescence. Solid lines: mean normalized fluorescence profiles across replicates. Shaded area, standard deviation across replicates. (D, G) The polar localization index measures the relative fraction of the fluorescence signal at the poles compared to the cytoplasm (cf. Materials and Methods). PilG always localizes predominantly polarly. PilH is mostly cytoplasmic at 0 h but becomes more polar over time. (E, H) Quantification of protein polarization. The asymmetry index measures the difference in fluorescent intensity between poles (cf. Materials and Methods). In (D), (E), (G), and (H), each circle corresponds to the population median of one biological replicate and the vertical bars to the mean across biological replicates. *P < 0.05; **P ≤ 0.001; ns, not significant (one‐way ANOVA and Tukey's *post hoc* test). For corresponding example images see Appendix Fig S1A. For corresponding fluorescence measurements see Appendix Fig S2A.

To investigate how Chp activates response regulators upon surface contact, we monitored localization of functional mNeonGreen‐tagged PilG and PilH as cells transition from liquid to a surface. Immediately after surface contact, which reflects the state of planktonic cells that have yet to mechanosense, mNG‐PilG predominantly localizes to the poles, while mNG‐PilH is mostly cytoplasmic (Fig 1A). Only a few cells display relatively dim PilH polar foci (Appendix Fig S1A). PilG displays pronounced asymmetrical localization, i.e., PilG is polarized (Fig 1A and B). We quantified subcellular localization profiles of hundreds to thousands of cells in each condition from which we generate population‐averaged fluorescent profiles (Fig 1C–F). With these, we derive a polar localization index measuring the ratio of polar versus cytoplasmic localization. We found that PilG polar localization is largely insensitive to time on the surface (Fig 1C and D). To quantify protein polarization (cf. Fig 1B), we defined an asymmetry index quantifying the intensity difference between the two polar spots. PilG is markedly and stably polarized, as the asymmetry index remains unchanged for 2 h on a surface (Fig 1E).

Unlike PilG, PilH is almost exclusively found in the cytoplasm early after surface contact but becomes increasingly localized to the poles after 1 and 2 h of surface contact (Fig 1F and G). While PilH polar localization is always lower compared to PilG, its localization index increases over time on surface (Fig 1G). PilH asymmetry index is steadily close to 0.5, i.e., nearly symmetric, showing PilH does not polarize (Fig 1H). In summary, PilG remains strongly polarized, only switching polarization during reversals (Kühn *et al*, 2021). PilH is largely cytoplasmic, but polar localization increases over time on surfaces without polarizing. Sustained PilG and PilH phosphorylation by ChpA during surface growth could potentially explain these changes in localization. We thus went on to identify the function of ChpA in polar localization of the two response regulators.

### ChpA induces PilG polarization through phosphorylation

**Figure 2:**
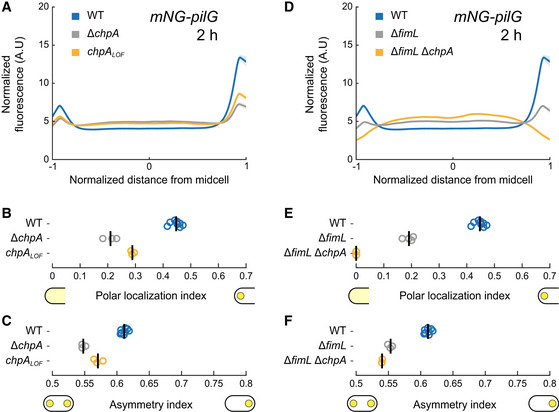
ChpA controls PilG polarization by phosphorylation A
Fluorescence profiles of mNG‐PilG in *chpA* mutants after 2 h on surfaces. Solid lines, mean normalized fluorescence profiles across biological replicates. Shaded area, standard deviation across biological replicates.B, C
(B) Polar localization index and (C) asymmetry index measurements of ChpA‐ and phosphorylation‐dependent localization of mNG‐PilG. Both deletion of *chpA* and inhibition of phosphorylation through ChpA reduce the polar localization index of PilG as well as polarization.D
Polar localization profiles of mNG‐PilG in Δ*fimL* and Δ*fimL* Δ*chpA* mutant after 2 h on surfaces.E, F
(E) Polar localization index shows that double deletion of *fimL* and *chpA* completely abolishes polar localization of PilG. Single deletion of *fimL* reduces but does not abolish PilG polar localization and polarization (F). Data information: Circles, median of each biological replicate. Vertical bars, mean across biological replicates. For corresponding mean cell fluorescence, see Appendix Fig S2B.

PilG preferentially localizes to the leading pole of twitching cells where T4P actively extend and retract (Kühn *et al*, 2021). We therefore hypothesize that in response to mechanosensing with T4P, the histidine kinase ChpA polarizes PilG by phosphorylation. To characterize the role of phosphorylation in PilG polarization, we compared the localization of mNG‐PilG between a *chpA* deletion mutant and wild type (WT). In Δ*chpA*, PilG polar localization decreases but is not entirely abolished (Fig 2A and B). The asymmetry index is also lower in Δ*chpA* compared to WT, showing ChpA promotes PilG polarization (Fig 2C). This is consistent with a model where ChpA promotes PilG polarization toward the leading pole in response to T4P input (Kühn *et al*, 2021).

To rigorously demonstrate that phosphorylation promotes PilG polarization, we interfered with the ability of the ChpA to signal to response regulators. Substituting three residues in the histidine kinase domain (D2086A, D2087A, and G2088A) blocks autophosphorylation, resulting in a loss‐of‐function (LOF) mutant. ChpA_LOF_ is unable to transfer phosphate to PilG and PilH (Bertrand *et al*, 2010; Silversmith *et al*, 2016) but still localizes to the cell poles (Appendix Fig S3A and B). Like Δ*chpA*, *chpA*_*LOF*_ mutants neither twitch nor increase intracellular cAMP levels on surfaces (Appendix Fig S4B; Bertrand *et al*, 2010). PilG polar localization and asymmetry indexes are much lower in *chpA*_*LOF*_ compared to WT (Fig 2B and C), showing that ChpA promotes PilG polarization by phosphorylation. ChpA_LOF_ may also weakly bind PilG independent of phosphorylation as PilG polar localization is slightly higher in *chpA*_*LOF*_ compared to Δ*chpA* (Fig 2B). We assessed whether *chpA* mutations could affect PilG localization due to cAMP‐dependent transcriptional changes. We quantified the localization and polarization of mNG‐PilG at low and high cAMP levels by respective deletion of the adenylate cyclase gene *cyaB* and phosphodiesterase gene *cpdA*. PilG localization was largely insensitive of cAMP levels (Appendix Fig S5). Our results therefore support a model in which T4P mechanosensing events at the leading pole stimulate ChpA to phosphorylate PilG to induce polarization.

PilG localization decreases but is not abolished in Δ*chpA* mutants, suggesting there exists an additional polar binding partner. We hypothesized that FimL, a protein that interacts with the polar hub FimV and PilG, could fulfill that role (Inclan *et al*, 2016). To characterize the impact of FimL on PilG polar localization, we imaged mNG‐PilG in a *fimL* deletion mutant. Polar localization and asymmetry of PilG decreased in Δ*fimL* compared to WT (Fig 2D–F). PilG polar localization was entirely abolished in a Δ*fimL* Δ*chpA* double‐deletion background (Fig 2E), showing that FimL and ChpA simultaneously recruit PilG to the poles. As in WT, loss‐of‐function mutation of *chpA* in Δ*fimL* background reduces PilG polar localization (Appendix Fig S6A and B). However, the asymmetry index is not affected by this mutation (Appendix Fig S6C). This indicates that FimL and ChpA must act together for correct PilG polarization.

Taken together, our results specify the molecular mechanisms driving PilG polar localization and polarization. Both FimL and ChpA are required for proper polarization of PilG. FimL maintains at least non‐phosphorylated PilG at the poles (Inclan *et al*, 2016). ChpA recruits PilG to transfer phosphate, thereby polarizing PilG and driving forward twitching (Kühn *et al*, 2021).

To verify the role of phosphorylation in PilG function and localization directly, we attempted to generate a gain‐of‐function mutant that mimics the phosphorylated conformation of PilG by substituting the catalytic aspartate 58 to glutamate (*pilG*_*D58E*_). We also generated an aspartate to alanine mutant, yielding the loss‐of‐function mutant *pilG*_*D58A*_. We assume PilG_D58A_ adopts the non‐phosphorylated conformation. We quantified their localization in WT (Appendix Fig S7), Δ*fimL* and *ΔchpA*. PilG_D58E_ as well as PilG_D58A_ are recruited to the poles by both FimL and ChpA similar to wild‐type PilG (Appendix Fig S8A–D). Both mutants, however, phenocopied Δ*pilG* in cAMP levels and twitching motility (Appendix Fig S9), indicating both mutants have lost function (we therefore refer to the amino acid substitutions for *pilG*, instead of *GOF* and *LOF*). These observations are inconsistent with the predicted mutational effects of *pilG*_*D58E*_. We thus failed to obtain the desired gain‐of‐function mutant by this method, whose limitations are however well‐documented (Smith *et al*, 2004; Guzzo *et al*, 2018). Our results suggest that PilG_D58E_ may have lost functionality due to a defective interaction with downstream targets or that phosphate transfer to and from PilG may be important.

### Polar recruitment of PilH depends on ChpA but not on its kinase activity

By analogy with PilG, phosphorylation by ChpA could explain the slow increase in PilH polar localization during surface association (Fig 1G). To test this possibility, we quantified mNG‐PilH localization in Δ*chpA* and *chpA*_*LOF*_. We first controlled the potential indirect effects of cAMP. In a Δ*cyaB* background with low cAMP, PilH polar localization was reduced compared to WT (Appendix Fig S10C). Conversely, polar localization was stronger in Δ*cpdA* with high cAMP levels. The difference in polar localization between WT and Δ*cpdA* leveled out after 2 h of surface contact, as both polar localization indexes became indistinguishable (Appendix Fig S10C). PilH localization is therefore sensitive to cAMP levels. To characterize the additional role of ChpA in PilH localization independently of cAMP, we performed all subsequent mNG‐PilH localization experiments in a Δ*cpdA* mutant background.

**Figure 3:**
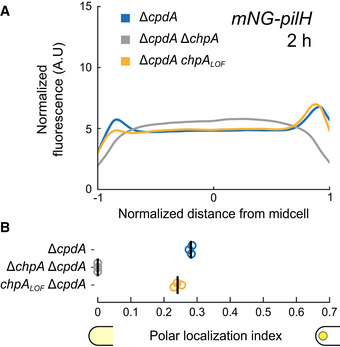
ChpA promotes PilH localization in a phosphorylation‐independent manner mNG‐PilH fluorescence profiles in *chpA* mutants after 2 h surface growth. To avoid negative effects of low cAMP level on localization of PilH, *cpdA* was deleted in all displayed strains to rescue cAMP level to WT levels (cf. Appendix Fig S4B). Solid lines, mean normalized fluorescence profiles across biological replicates. Shaded area, standard deviation across biological replicates.PilH polar localization is abolished in Δ*chpA*. PilH polar localization is however maintained in *chpA*_*LOF*_. Circles, median of each biological replicate. Vertical bars, mean across biological replicates. For corresponding asymmetry indexes and mean cell fluorescence, see Appendix Fig S2C.

mNG‐PilH polar localization is abolished in Δ*chpA* Δ*cpdA* (Fig 3A and B), despite nearly identical protein levels as in Δ*cpdA* (Appendix Fig S2C) and high cAMP levels (Appendix Fig S4B). Polar localization of PilH is therefore absolutely ChpA‐dependent. To address whether PilH localization depends on phosphorylation, we imaged mNG‐PilH in the non‐functional *chpA*_*LOF*_ mutant with rescued cAMP level (Δ*cpdA* background). The polar localization index of PilH in *chpA*_*LOF*_ Δ*cpdA* is close to the one in Δ*cpdA* (Fig 3B). These results show that despite depending on ChpA, polar recruitment of PilH does not depend on phosphorylation through ChpA. It is therefore possible that both non‐phosphorylated and phosphorylated forms of PilH localize to the poles. We found that cAMP levels partially control polar localization of PilH. The slow increase in cAMP levels during surface contact (Persat *et al*, 2015a) could thereby explain the increasing polar localization of PilH over time (Fig 1G).

Analogous to PilG, we generated a PilH mutant that cannot be phosphorylated by substituting the catalytic aspartate 52 with alanine to make *pilH*_*LOF*_ (LOF, loss‐of‐function). *pilH*_*LOF*_ cells twitch forward without reversing and have elevated cAMP levels similar to Δ*pilH* mutants, confirming the loss‐of‐function mutation (Appendix Fig S11). Substituting aspartate 52 to glutamate yields a gain‐of‐function (GOF) mutant, *pilH*_*GOF*_, which is predicted to adopt an active phosphorylated conformation but loses the ability to be phosphorylated. Reversal rate measurements and cAMP levels confirmed that PilH_GOF_ is functional and can complement a deletion of *pilH*, although both phenotypes are not quantitatively restored to WT levels (Appendix Fig S11).

**Figure 4:**
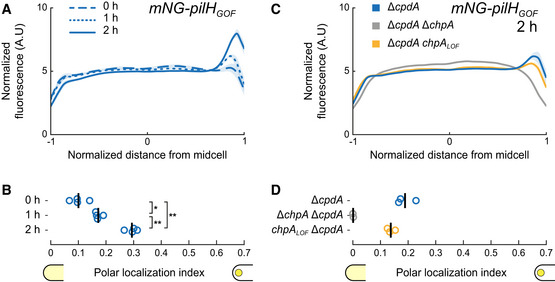
ChpA recruits active PilH to the pole in a phosphorylation‐independent manner Time‐course fluorescence profiles of mNG‐PilH_GOF_.Like wild‐type PilH, PilH_GOF_ gets recruited to the poles over time on the surface.Fluorescence profiles of mNG‐PilH_GOF_ after 2 h on surface in *chpA* mutants.Polar localization index measurements show that polar localization of mNG‐PilH_GOF_ depends on ChpA, but not on its kinase activity. Data information: (A, C) Solid lines, mean normalized fluorescence profiles across biological replicates. Shaded area, standard deviation across biological replicates. (B, D) Circles, median of each biological replicate. Vertical bars, mean across biological replicates. *P < 0.05; **P ≤ 0.001; ns, not significant (one‐way ANOVA and Tukey's *post hoc* test). For corresponding asymmetry indexes and mean cell fluorescence see Appendix Fig S2D and E.

Like wild‐type PilH, PilH_GOF_ slowly increases polar localization (Fig 4A and B). PilH_LOF_ also slowly localizes to the poles but less pronounced than PilH or PilH_GOF_ (Appendix Fig S12). The slow recruitment in both PilH mutants and wild‐type PilH is consistent with a scenario where polar localization of PilH is independent of phosphorylation. Like for wild‐type PilH, polar localization of PilH_GOF_ is ChpA‐dependent but independent of ChpA's ability to autophosphorylate and transfer phosphate (Fig 4C and D). This suggests that ChpA recruits both PilH and activated PilH‐P to the poles. Although direct interaction between PilH forms and ChpA has yet to be demonstrated, interactions between response regulators and histidine kinases are well‐established in homologous chemosensory systems (Kentner & Sourjik, 2009). Regarding Chp, *in vitro* phosphorylation of purified PilG and PilH by ChpA strongly suggests direct interaction of ChpA with both PilG and PilH (Silversmith *et al*, 2016).

### The signaling hierarchy between PilG and PilH impacts twitching

**Figure 5:**
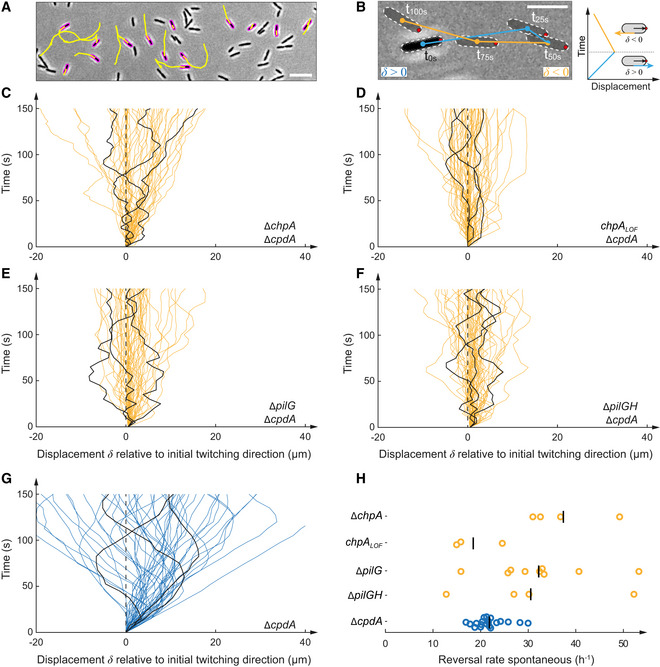
ChpA regulates forward and reverse twitching A
Snapshot of 5‐min‐long twitching trajectories (yellow lines). Scale bar, 10 μm.B
Computation of displacement maps and reversal rates. A change in the orientation of the track relative to the initially leading pole (red dot) corresponds to a reversal. Scale bar, 5 μm.C–G
Displacement maps derived from 50 randomly selected cell trajectories from three random biological replicates. Each curve corresponds to an individual trajectory. A curve oriented to the top right corresponds to forward twitching, and a curve oriented to the top left corresponds to reverse twitching. In each graph, we highlighted three representative tracks for clarity.H
Corresponding spontaneous reversal rates. To bypass low cAMP levels, *cpdA* was deleted in all displayed strains. We use Δ*cpdA* as reference. Deletion and loss‐of‐function mutation of *chpA* show a hyper‐reversing phenotype, similar to deletion of *pilG*. Δ*pilG* Δ*pilH* double mutants also hyper‐reverse, phenocopying Δ*pilG* and Δ*chpA*/*chpA*_*LOF*_ mutants. For corresponding example movies, see Movie EV2.

The dynamic polarization of PilG during motility and localization of PilH during surface adaptation motivated us to investigate the role of phosphorylation during twitching. In response to mechanosensing, Chp controls forward twitching by polarizing PilB while still enabling spontaneous and collision‐induced reversals (Kühn *et al*, 2021). PilG is necessary for forward twitching, while PilH is required for reversals (Kühn *et al*, 2021). We tracked hundreds of isolated motile cells twitching at the interface between agarose and a glass coverslip (Fig 5A and B). We computed their linear displacements, which we display as spatial–temporal diagrams (Fig 5C–G), and we calculated reversal frequencies (Fig 5H). We had previously shown that *pilG* deletion mutants constitutively reverse, while *pilH* deletion mutants always move forward without reversing (Kühn *et al*, 2021). Here, we found that Δ*chpA* and *chpA*_*LOF*_ mutants constantly reversed twitching direction (Fig 5C, D and H, Movie EV2), qualitatively recapitulating the hyper‐reversing phenotype of Δ*pilG* (Fig 5E and H, Movie EV2). The inability to phosphorylate PilG and PilH thus phenocopies Δ*pilG*, not Δ*pilH*. Therefore, phosphorylation of at least PilG is required to induce persistent forward twitching.

We wondered whether PilH itself was required to trigger reversals in mutants that are already hyper‐reversing. The double‐deletion mutant Δ*pilG* Δ*pilH* also hyper‐reverses (Fig 5F and H, Movie EV2), phenocopying Δ*pilG*. This demonstrates that PilH is not inherently required to reverse. PilH may only be required for reversals when PilG is activated by phosphorylation, i.e., when cells are already polarized. Our results suggest that PilH does not trigger reversals by directly inhibiting polarization of the extension motor PilB (Kühn *et al*, 2021). PilH may instead interfere with PilG's function in maintaining a positive feedback that promotes T4P polarization. This finding suggests PilG is the main output regulator of the Chp phosphorylation cascade, controlling directionality of twitching, and that PilH functions antagonistically by counteracting PilG.

### PilG does not directly regulate PilH localization upon surface contact

**Figure 6:**
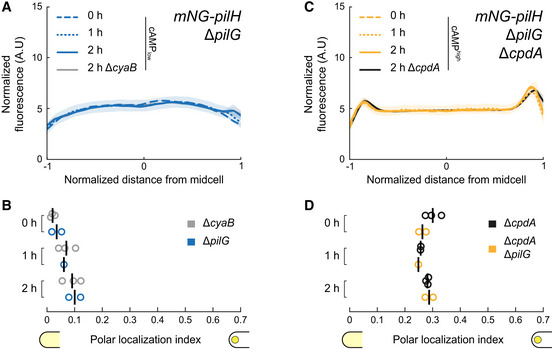
Localization of PilH is not directly affected by PilG mNG‐PilH fluorescence profiles in Δ*pilG* after 2 h surface growth (low cAMP).Corresponding polar localization index of mNG‐PilH in Δ*pilG*. Recruitment of PilH to the poles is slow but detectable in Δ*pilG*, almost identical to Δ*cyaB* which has similar cAMP levels (Appendix Fig S4A).mNG‐PilH fluorescence profiles in Δ*pilG* Δ*cpdA* after 2 h surface growth (rescued cAMP).Corresponding polar localization index of mNG‐PilH in Δ*pilG* Δ*cpdA*. PilH polar recruitment is indistinguishable from Δ*cpdA*, which also has elevated cAMP levels (Appendix Fig S4A). Solid lines, mean normalized fluorescence profiles across biological replicates. Shaded area, standard deviation across biological replicates. Circles, median of each biological replicate. Vertical bars, mean across biological replicates. For corresponding asymmetry indexes and mean cell fluorescence see Appendix Fig S2F.

To further refine the mechanisms of PilG's and PilH's antagonistic relationship, we investigated how they impact each other's localization. We could not distinguish a change in mNG‐PilH localization in a Δ*pilG* mutant background, supporting that PilG does not regulate PilH activation (Fig 6A and B). Since PilH localization is sensitive to cAMP levels, we confirmed PilG‐independent localization in Δ*pilG* Δ*cpdA*, whose cAMP levels are rescued to near WT levels (Fig 6C and D).

### PilH activation controls PilG polarization

**Figure 7:**
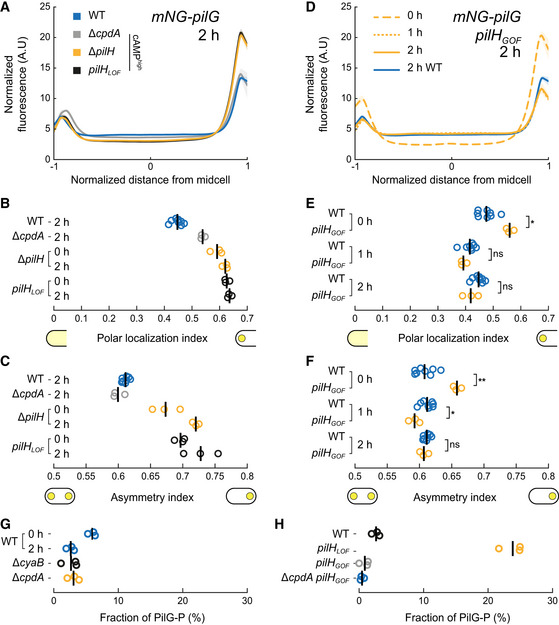
PilH activation modulates PilG polar localization A–F
(A) mNG‐PilG fluorescence profiles in *pilH* mutants after 2 h surface growth. We included Δ*cpdA* for comparison since both *pilH* mutations result in high cAMP levels. PilG is more polar and asymmetric in Δ*pilH* and *pilH*_*LOF*_. (B) Polar localization index and (C) asymmetry index measurements of PilH‐dependent localization of mNG‐PilG. (D) Time course of PilG localization in *pilH*_*GOF*_ on the surface. For clarity, only the 2 h profile of PilG in wild type from Fig 1C was included. (E, F) Corresponding polar localization and asymmetry index measurements. In *pilH*_*GOF*_, polar localization of PilG is comparable to Δ*pilH* and *pilH*_*LOF*_ at 0 h, but similar to wild type after 1 and 2 h surface growth. (A and D) Solid lines, mean normalized fluorescence profiles across biological replicates. Shaded area, standard deviation across biological replicates. (B, C, E, F) Circles, median of each biological replicate. Vertical bars, mean across biological replicates. *P < 0.05; **P ≤ 0.001; ns, not significant (one‐way ANOVA and Tukey's *post hoc* test). For corresponding mean cell fluorescence see Appendix Fig S2G. For corresponding example micrographs see Appendix Fig S1B.G
PhosTag™ measurements of the fraction of phosphorylated Flag‐PilG (PilG‐P) after 2 h surface growth. For WT we also show 0‐h data. The fraction of PilG‐P decreases slightly on surfaces, consistent with slightly decreasing PilG polar localization upon surface contact (cf. Fig 1C and D).H
Fraction of phosphorylated PilG after 2 h surface growth in *pilH*_*GOF*_ and *pilH*_*LOF*_. To rule out a negative effect of lower cAMP level in *pilH*_*GOF*_, we also deleted *cpdA* in that mutant. Circles correspond to biological replicates, and black bars represent their mean. See Appendix Fig S14 for a representative PhosTag™ gel.

PilH may function antagonistically by directly inhibiting PilG. To characterize how PilH impacts PilG polarization, we quantified PilG localization in Δ*pilH*. Polar localization of PilG is increased in Δ*pilH* relative to WT and to Δ*cpdA* whose cAMP levels nearly match Δ*pilH* (Fig 7A and B; Kühn *et al*, 2021). In addition, the asymmetry index of mNG‐PilG is greater in Δ*pilH* than in WT (Fig 7C). Accordingly, mNG–PilG polar localization index and asymmetry index in *pilH*_*LOF*_ are indistinguishable from Δ*pilH* (Fig 7B and C). We conclude that PilH represses PilG polar localization and polarization.

We wondered whether PilH activation by ChpA is necessary to repress PilG polarization. To decouple PilH activation from ChpA and allow native PilG activation by ChpA at the same time, we measured PilG localization in *pilH*_*GOF*_ (Fig 7D–F). PilG polar localization and asymmetry in *pilH*_*GOF*_ at 0 h are much closer to Δ*pilH* and *pilH*_*LOF*_ than to WT (cf. Fig 7A–C vs. D–F). Both indexes however decrease over time on the surface to eventually reach WT levels. This is consistent with a model where PilH in its active conformation reduces the polar localization of PilG and thus mitigates PilG polarization. This effect is independent of PilH‐related phosphate flow since PilH_GOF_ cannot get phosphorylated. In addition, this only takes effect after surface contact, which recruits PilH and PilH_GOF_ to the poles, and is not an inherent property of PilH_GOF_.

In summary, PilH is required for proper localization of PilG. Without PilH, PilG localization is extremely polarized, likely irreversible, explaining the unidirectional twitching phenotype observed in Δ*pilH* (Kühn *et al*, 2021). Upon recruitment to the poles, PilH in its active conformation reduces polar localization of PilG. Since PilH and PilH_GOF_ polar localization are ChpA‐dependent, we conclude that PilH functions through ChpA. We verified that PilH requires functional ChpA but not FimL to modulate PilG polar localization (Appendix Fig S13). PilH locked in its active conformation, PilH_GOF_, is sufficient to reduce PilG localization. This process does not require phosphate transfer involving PilH, disproving the hypothesis that PilH acts as a phosphate sink for PilG and ChpA (Fulcher *et al*, 2010; Silversmith *et al*, 2016).

### PilH activation impacts PilG phosphorylation

Since phosphorylation stimulates PilG polarization, PilH could indirectly regulate PilG polarization by controlling PilG phosphorylation. We thus measured the fraction of phosphorylated PilG in whole‐cell lysates by PhosTag™ assays in *pilH*_*LOF*_, which causes strong polarization, and in *pilH*_*GOF*_ which has WT‐like polar localization. First, we verified that Flag‐tagged PilG migrated as two bands on PhosTag™ gels (Appendix Fig S14A). The slower migrating band was absent in *pilG*_*D58A*_ and *pilG*_*D58E*_ point mutants that cannot be phosphorylated, suggesting that it represents phosphorylated PilG. Since *pilH*_*LOF*_ and *pilH*_*GOF*_ mutations affect cAMP production, we verified that cAMP levels do not affect phosphorylation of PilG by measuring PilG‐P in WT, Δ*cyaB* (low cAMP) and Δ*cpdA* (high cAMP; Fig 7G). In *pilH*_*LOF*_, the fraction of phosphorylated PilG is higher than in WT (Fig 7H). The fraction of phosphorylated PilG‐P is relatively reduced in *pilH*_*GOF*_ and in *pilH*_*GOF*_ Δ*cpdA* (Fig 7H). This suggests PilH in its phosphorylated conformation reduces PilG phosphorylation without the need of phosphate flow to or from PilH. PilG phosphorylation decreases slightly on surfaces (Fig 7G), possibly due to PilH polar recruitment.

To test the model where PilH regulates PilG localization by controlling PilG phosphorylation, we quantified PilH‐dependent localization of non‐phosphorylatable PilG_D58A_ and PilG_D58E_, despite the decreased functionality of PilG_D58E_. Localization of both mutants in Δ*pilH* is indistinguishable from WT (Appendix Fig S15). Accordingly, *pilH* deletion has no effect on twitching of *pilG*_*D58A*_ and *pilG*_*D58E*_ (Movie EV3). This confirms that PilH functions through modulation of PilG phosphorylation.

### Mechanosensing shifts the balance of an ongoing competition between PilG and PilH

**Figure 8:**
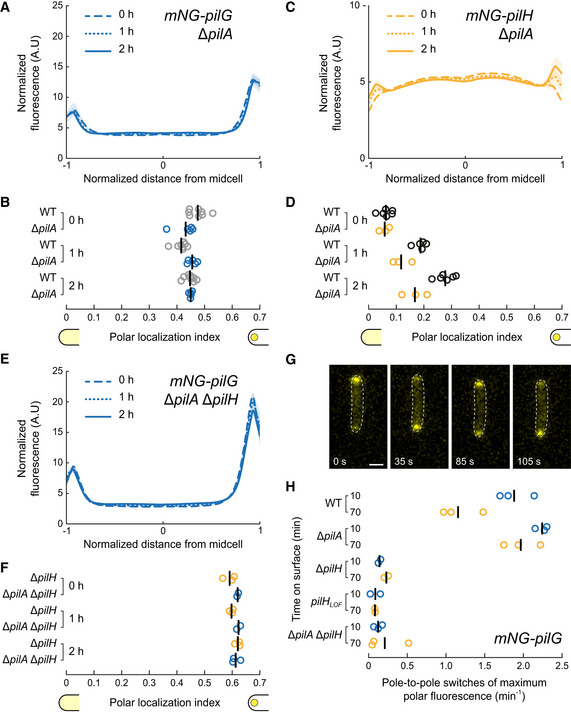
Mechanosensing though PilA and Chp controls dynamic PilG localization A, B
Time course fluorescence profiles and polar localization indexes of mNG‐PilG in Δ*pilA*. PilG polar localization index remains unchanged on surfaces in Δ*pilA* similar to surface‐adapted WT (WT data from Fig 1D).C, D
Time course fluorescence profiles and polar localization indexes of mNG‐PilH in Δ*pilA*. Like in WT, PilH gets recruited to the poles over time in Δ*pilA*, although at a slower speed (WT data from Fig 1G).E, F
Time course fluorescence profiles and polar localization indexes of mNG‐PilG in Δ*pilA* Δ*pilH* (WT data from Fig 7B). Deletion of *pilH* affects localization of PilG despite the lack of signal input from PilA. For corresponding asymmetry indexes and mean cell fluorescence see Appendix Fig S17.G
Snapshots of a 3 min long movie (Movie EV4) of mNG‐PilG moving from pole to pole shortly after surface contact. Scale bar, 1 μm.H
Quantification of the rate of pole‐to‐pole switches of the maximum mNG‐PilG signal. In Δ*pilA*, the rate remains high over time. Without PilH, independently of PilA, PilG is locked at one pole.

We found that Chp stimulates PilG polarization, indicating that surface sensing should stimulate PilG localization. However, we observed that PilG does not change polarization during surface contact (Fig 1E). We therefore wondered how mechanical stimuli from T4P could impact Chp‐dependent mechanotransduction to control reversals. To achieve this, we quantified PilG and PilH localization in Δ*pilA*. We found little difference in the overall localization patterns of response regulators between WT and Δ*pilA*. For example, PilG still localizes asymmetrically to the poles in Δ*pilA* (Fig 8A and B). In WT, the polar localization decreased slightly upon surface contact, whereas in Δ*pilA* PilG polar localization remains unchanged at the level of surface‐adapted WT. PilH and PilH_GOF_ still get recruited to the poles upon surface contact despite the lack of PilA, albeit at a slower speed (Fig 8C and D, Appendix Fig S16A and B). In Δ*pilA* Δ*pilH*, polar localization of PilG is comparable to Δ*pilH* (Fig 8E and F), indicating that PilH still regulates PilG independently of signal input from PilA, for example, in liquid. Accordingly, PilH_GOF_ still represses PilG polar localization in Δ*pilA*; however, the effect is slightly delayed (Appendix Fig S16E and F).

While the static localization pattern of PilG is not affected by *pilA* deletion, we wondered whether dynamic localization of PilG requires signal input from PilA. As does the extension machinery, we found that PilG oscillates from pole to pole right after surface contact and only later stably polarizes (Fig 8G and H). The rate of PilG polarization switches decreases after 1 h on surface in WT as it did for FimX (Kühn *et al*, 2021). However, under the same conditions, PilG sustains oscillations in a Δ*pilA* background. This suggests that mechanical input from T4P does not impact PilG polar localization quantitatively but rather locks polarization in the direction of mechanical input. This result further confirms that PilG polarization responds to a spatially defined mechanical input from T4P (Kühn *et al*, 2021).

Consistent with this, PilG is locked at one pole in Δ*pilH* and *pilH*_*LOF*_ (Fig 8H). In the double‐deletion mutant Δ*pilA* Δ*pilH*, PilG is also locked at one cell pole, showing that PilH is required for PilG dynamic movement from pole to pole. While the competitive interaction of PilG and PilH is likely occurring at all times, our results support a model in which mechanosensing through T4P shifts the balance of PilG and PilH signaling between poles.

## Discussion

**Figure 9:**
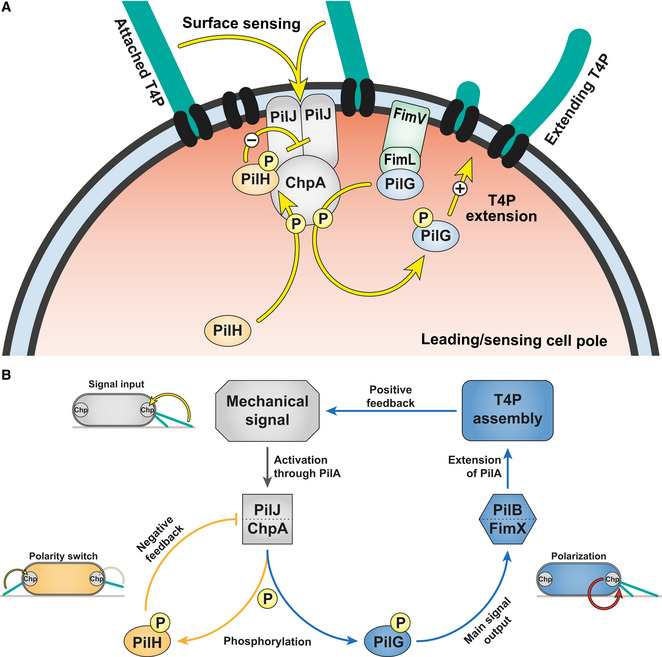
A model of signaling feedback in *Pseudomonas aeruginosa* mechanotaxis Model of phosphate flow through Chp at the leading pole of twitching cells. PilG is recruited by two polar proteins, ChpA and FimL. FimL maintains PilG close to Chp, potentially to restrict diffusion. ChpA phosphorylates PilG to promote and stabilize PilG polarization. PilG locally recruits FimX and PilB to promote T4P extension (Kühn *et al*, 2021). Polar localization of PilH is independent of phosphorylation. However, a conformational change induced by phosphorylation is required to activate PilH. PilH‐P mitigates the polarizing effect of PilG by reducing PilG phosphorylation.Overview of the Pil‐Chp regulation circuit. Chp senses a local mechanical signal when T4P attache at the leading pole of motile cells. Surface sensing involves interaction of T4P filament monomers PilA and Chp's receptor PilJ (Persat *et al*, 2015a; Koch *et al*, 2022). As a consequence, ChpA becomes more active and phosphorylates PilG. PilG constitutes the main signal output of Chp, eventually polarizing cells by recruiting FimX and PilB to the actively sensing pole where they locally activate T4P extension. As T4P themselves are the surface sensor, this creates a positive feedback loop. ChpA also phosphorylates PilH to balance the main signal output by modulating PilG's activity and localization. PilH achieves this by decreasing PilG phosphorylation, potentially by inhibiting PilJ/ChpA. Presumably, the activity of ChpA and thus the local balance of PilG and PilH differs between sensing and non‐sensing poles. Arrows depict hypothesized functional interactions, not direct protein–protein interactions.

How cells establish and switch polarity are critical questions in biology. Polarity is an essential requirement of the physiology of many bacteria. For example, cells polarize by asymmetric localization of cellular components during motility or asymmetric division (Treuner‐Lange & Søgaard‐Andersen, 2014). Our results bring a high‐resolution perspective on the Pil‐Chp signaling network controlling reversible polarity in *P. aeruginosa* in response to mechanosensing (Fig 9A and B). In particular, we identified the role of PilG and PilH activation in relaying information from mechanical input to a motility response. Cells sense surface contact through T4P. During attachment and retraction, T4P activate Chp through the receptor PilJ (Persat *et al*, 2015a; Koch *et al*, 2022). In a simple model, PilJ stimulates ChpA autophosphorylation at that pole. Surface sensing may also selectively activate some of ChpA's many phosphotransfer domains, while others may even be deactivated. ChpA then recruits PilG to transfer phosphate (Silversmith *et al*, 2016), thereby polarizing the cell. This in turn stimulates T4P extension via the recruitment of at least FimX and PilB (Kühn *et al*, 2021). Increased T4P deployment locally stimulates mechanosensing at that same pole, feeding a local positive feedback, which stabilizes polarization and forward motility. We showed that PilG phosphorylation by ChpA promotes polarization. PilG is however similarly polarized in liquid or without PilA‐dependent signal input. PilG and PilH phosphorylation may thus be an ongoing process both in liquid and on surfaces, and surface sensing merely shifts the activation balance between PilG and PilH at one of the poles. Consistent with this scenario, we detected a slight decrease in PilG‐P on surfaces (Fig 7G). Although PilG is always polarized, the direction of polarity frequently switches in cells that do not mechanosense surfaces (Fig 8G and H). PilA‐dependent surface sensing at one pole then stabilizes PilG polarization over time to enable persistent forward motility. This supports a model in which local activation by T4P promotes and stabilizes cell polarity, establishing the most direct molecular link between mechanical input (T4P retraction on surfaces) and cellular output (PilG polarization). Setting polarity only requires PilG, supporting the hypothesis that PilG is the main output regulator of the Chp system (Bertrand *et al*, 2010; Fulcher *et al*, 2010; Silversmith *et al*, 2016).

Local activation of PilG alone cannot explain asymmetric activation of T4P extension. In *E. coli*, CheY‐P quickly diffuses to flagellar basal bodies located throughout the cell envelope after phosphorylation (Maddock & Shapiro, 1993; Thiem *et al*, 2007; Briegel *et al*, 2009). Conceivably, cells need to restrict diffusion of PilG‐P to limit the positive feedback to the leading input pole. This could be achieved by either limiting phosphorylation to the signal input pole coupled with a short half‐life of PilG‐P or by restricting diffusion of PilG once phosphorylated. We postulate FimL, which is maintained at the poles by interaction with the polar landmark protein FimV (Inclan *et al*, 2016; Carter *et al*, 2017), could be involved in restricting PilG to the mechanosensing input pole (the leading pole in motile cells). This may restrict PilG‐P diffusion toward the opposite pole, while dynamic polarization of PilG‐P requires other factors such as phosphorylation. Interestingly, the N‐termini of FimL and ChpA are homologous, and both proteins are required for proper polarization of PilG, suggesting a connection between the two proteins, for example, in interacting with PilG (Inclan *et al*, 2016). FimL is further involved in phosphorylation‐independent asymmetry of PilG, although it is not as pronounced as phosphorylation‐dependent asymmetry (Fig 2). While the feedback‐driven asymmetry established by phosphorylation of PilG enables dynamic switching of polarity in response to mechanical signal input, the role of FimL‐dependent localization of PilG remains to be clarified in future studies.

Local positive feedback promotes forward locomotion but prevents directional changes. Without a mechanism to counteract this feedback, motile cells would trap themselves in corners, and cell groups would jam during collective twitching (Kühn *et al*, 2021; Meacock *et al*, 2021). *P. aeruginosa* avoids locking itself into one movement direction by mitigating the positive feedback. At the molecular level, the response regulator PilH counteracts PilG to invert polarization and enable reversals of twitching direction. Multiple conflicting molecular models have been proposed for the antagonizing effect of PilH (Bertrand *et al*, 2010; Fulcher *et al*, 2010; Silversmith *et al*, 2016). Here, we resolved the mechanism of PilH function in the Chp signaling system. We show that PilH directly regulates PilG polarization by reducing PilG phosphorylation. To reverse PilG polarization, PilH must transition to its active conformation, for which it must be phosphorylated. PilH‐P may diffuse across the cytoplasmic space to create a global antagonistic effect at both poles, or PilH and PilH‐P may localize to different poles generating a polarized antagonistic effect. This in turn interrupts the positive feedback perpetuated by PilG. In WT, PilH and PilH‐P localize to the poles without apparent polarization, making the investigation of PilH dynamic activation in motile cells complex. Both PilH and constitutively activated PilH_GOF_ require ChpA for polar localization and for regulating PilG. This indicates that PilH interacts with ChpA, and that once phosphorylated PilH‐P inhibits PilG phosphorylation by ChpA, potentially by blocking or inhibiting the phosphotransfer domain(s) responsible for PilG phosphorylation. PilH‐P may also stimulate the receiver domain of ChpA (ChpA_REC_) that may function as a phosphate sink for PilG. The autodephosphorylation rate of ChpA_REC_‐P is order of magnitudes faster than of PilG‐P or PilH‐P, consistent with a sink function (Silversmith *et al*, 2016).

Polar localization of PilH gradually increases during surface adaptation, however, independently from PilH phosphorylation or PilA. Instead, polar recruitment of PilH correlates well with cAMP levels (cf. Appendix Figs S4A and S10C), suggesting PilH localization is regulated by transcription (Beatson *et al*, 2002; Wolfgang *et al*, 2003). However, PilH localization does not correlate with mNG‐PilH protein abundance (cf. Appendix Fig S10C and E), and PilH recruitment is only delayed by constitutively low cAMP levels in Δ*cyaB* or Δ*pilA*. It is therefore unlikely that protein expression alone explains PilH polar recruitment. As both PilH and PilH_GOF_ require ChpA for polar localization, the factors responsible for recruiting PilH to the pole may at least involve interaction with ChpA. In contrast, despite increasing polar recruitment of PilH, PilG polar localization and polarization are not considerably affected by surface sensing on a long timescale, indicating some level of homeostasis. Polarization changes of PilG on the other hand are considerably affected by PilA‐dependent surface sensing and require functional PilH. This hints at a very intricate local and dynamic interplay of PilG and PilH phosphorylation. Resolving phosphorylation of these response regulators within a cell would provide invaluable information to resolve these connections, but this remains a technical challenge with current methods.

Chp‐like systems with multiple response regulators may be involved in surface‐induced and taxis‐related phenotypes in other species. Chp homologs are present in a number of bacterial species, for example, many gamma‐proteobacteria besides *P. aeruginosa*, including *Acinetobacter baumanni*, *Stenotrophomonas maltophilia*, or *Dichelobacter nodosus*, among others (Inclan *et al*, 2016). In *Acinetobacter* species, T4P mediate surface motility and natural transformation. ChpA and PilG are required for motility and transformability in *A. baumanii* (Vesel & Blokesch, 2021) and motility in *A. baylyi* (Leong *et al*, 2017). In *A. baylyi*, PilG localizes on a line oriented along the cell body axis, a mechanism that depends on FimL (Ellison *et al*, 2022). PilH may also function antagonistically to PilG as in *P. aeruginosa* (Vesel & Blokesch, 2021).

The cyanobacterium *Synechocystis* uses phototaxis to direct T4P‐dependent surface motility. Several chemotaxis‐like systems are involved, three of which encode two response regulators as in Chp (Han *et al*, 2022). The response regulators PixG, PilG, and TaxP2 have an N‐terminal PATAN domain that is missing in PilG of *P. aeruginosa* and related systems. The PATAN domain mediates direct interaction with the T4P extension motor PilB, whose localization sets twitching direction in response to light (Schuergers *et al*, 2015, 2016; Han *et al*, 2022). The output functions of their second response regulators PixH, PilH, and TaxY2 have yet to be resolved, but based on our results in *P. aeruginosa*, one could anticipate that they counter the function of their cognate first response regulator. In *Myxococcus xanthus*, a chemotaxis‐like system called Frz regulates the frequency of motility reversals, in the same manner as Chp in *P. aeruginosa*. Frz controls the localization of MglA, MglB, and RomR to the leading or lagging pole, thereby setting the direction of gliding and twitching motility (Blackhart & Zusman, 1985; Kaimer & Zusman, 2016; Guzzo *et al*, 2018; Szadkowski *et al*, 2019; Carreira *et al*, 2022). Here, the output response regulators FrzX and FrzZ show clear localization to opposite poles, in contrast to PilG and PilH (Guzzo *et al*, 2018).

Although Chp comprises a more complex signaling network than the dominant model of chemotaxis systems, the Che system controlling flagellar rotation in *E. coli*, there are even more complex chemosensory systems in many species. They often comprise several copies of chemosensory components, including response regulators, but also of multiple sets of full chemotaxis systems. Why some species require such complexity in chemotaxis systems remains poorly understood. In *Rhodobacter sphaeroides*, multiple chemotaxis systems control flagellar rotation in response to different signal inputs (Porter *et al*, 2011). Remarkably, only one main response regulator actively stops the flagellar motor, while others may only modulate the effect of the main regulator, indicating a shared signaling principle with Chp (Beyer *et al*, 2019).

We here decoded the signaling pathway of the Chp system during mechanotaxis. Our results resolve the molecular mechanisms of signaling with two response regulators. At the same time, our model proves a rationale for the complexity of Chp by demonstrating the necessity of each component in setting a local positive feedback while still enabling reversals. Our data therefore provide a new framework to understand more complex sensory systems in bacteria.

## Materials and Methods

### Bacterial strains, growth conditions, and media

For all experiments, *P. aeruginosa* PAO1 ATCC 15692 (American Type Culture Collection) was used. For vector constructions and conjugative mating, *E. coli* strains DH5α and S17.1 were used, respectively. All bacterial strains were grown in LB medium (Carl Roth) at 37°C with 280 rpm shaking. Regular solid LB media were prepared by adding 1.5% (wt · vol^−1^) agar (Fisher Bioreagents) and appropriate antibiotics for selection of *E. coli* 100 μg ml^−1^ ampicillin or 10 μg ml^−1^ gentamycin and for selection of *P. aeruginosa* 300 μg ml^−1^ carbenicillin or 60 μg ml^−1^ gentamycin. For twitching and protein localization experiments, semi‐solid tryptone media were prepared by autoclaving (5 g l^−1^ tryptone (Carl Roth), 2.5 g l^−1^ NaCl (Fisher Bioreagents), 0.5% (wt vol^−1^) agarose standard (Carl Roth)). For measurements of cAMP levels on solid surfaces, LB plates containing 1% standard agarose were prepared by autoclaving (for the PaQa reporter), or regular 1.5% LB agar plates were used (for the PlacP1 reporter). Surface growth for PhosTag™ assays was carried out on regular 1.5% LB agar plates.

### Strains and vector construction

Strains are listed in Appendix Table S1, and plasmids and corresponding oligonucleotides are listed in Appendix Tables S2 and S3, respectively. *Pseudomonas aeruginosa* mutants were generated as described previously (Kühn *et al*, 2021). Genes were deleted or integrated by two‐step allelic exchange according to (Hmelo *et al*, 2015) using the suicide vectors pEX18_Amp_ or pEX18_Gent_. For genomic in‐frame gene deletions, approximately 500–1,000 base‐pair fragments of the up‐ and downstream regions of the designated gene were combined by PCR amplification and subsequent Gibson assembly (Gibson *et al*, 2009). Marker‐free deletions were verified by PCR and sequencing. In‐frame insertions were generated essentially the same way. Substitutions of wild‐type genes with mutated genes (e.g., point mutants) were integrated into the corresponding deletion strains or wild type. Proteins were fluorescently labeled typically by N‐terminal fusion separated by a 5xG linker and integrated into the wild‐type gene locus. Functionality was tested by monitoring single‐cell twitching and measurements of cAMP levels (see also Kühn *et al*, 2021). Plasmids were constructed using standard Gibson assembly protocols (Gibson *et al*, 2009) and introduced into *P. aeruginosa* cells by conjugative mating with *E. coli* S17.1 as the donor.

### Fluorescence microscopy

Microscopy was performed on an inverted Nikon TiE epifluorescence microscope using NISElements (version AR 5.02.03). For phase contrast microscopy, a 40× Plan APO NA 0.9 phase contrast objective was used. For fluorescence microscopy, a 100× Plan APO NA 1.45 phase contrast oil objective and Semrock YFP‐2427B or TxRed‐A‐Basic‐NTE filters were used as needed. Microscope settings were kept consistent throughout all experiments to ensure comparability of fluorescent intensities. Fluorescent images were background‐subtracted and snapshots and movies were generated with ImageJ (version 1.53). Data were analyzed with custom scripts using Python (version 3.8.5) and MATLAB (version R2019b), as specified in detail below. Custom codes are available on Github (https://github.com/PersatLab/antagonists).

### Media and cell preparation for single‐cell twitching experiments

Plates were prepared by autoclaving tryptone medium supplemented with 0.5% agarose, cooling to 70°C in the autoclave followed by cooling to 55°C for 20 min in a water bath. In total, 28 ml medium was poured into 90‐mm petri dishes and dried in a flow hood for 30 min. Plates were always stored for 1 day at 4°C in a plastic bag and used the next day or maximum after 2 days. Exponentially growing cells (filtered LB medium, OD_600_ = 0.2–0.8) were diluted to OD_600_ = 0.2. After prewarming the plates for 45–60 min a 16‐mm round pad was cut out and 1 μl of the diluted cell suspension was pipetted onto the upper side of the agarose pad (i.e., the side that was not in contact with the plastic dish bottom). The pads were immediately flipped onto a microscope glass bottom dish (P35G‐1.5‐20‐C, MatTek) and four droplets of PBS were added at the sides without touching the pad to prevent drying. The dishes were used for imaging immediately or incubated at 37°C for later imaging.

### Quantification of protein localization

All displayed datapoints correspond to individually repeated biological replicates. Cells were prepared and imaged as described above. Although protein localization was analyzed independent of twitching direction, motile cells were imaged. Motile cells are typically visible after 1 and 2 h but not at 0 h. The analysis was carried out essentially as described previously (Kühn *et al*, 2021). Fluorescent images of mNeonGreen fusion proteins were acquired at 0, 1, and 2 h after preparation of the microscope dishes. 0‐h samples were taken within 1–5 min after the cell suspension was pipetted onto the tryptone agarose pads. Imaging settings (objective and filters, excitation power and time, binning) were kept consistent across all experiments to ensure comparability of the fluorescent signal. Cells were segmented using phase contrast images, and fluorescence profiles were extracted with BacStalk (version 1.8, Hartmann *et al*, 2020). Typically, several dozens to hundreds of cells were segmented per replicate, for total numbers of segmented cells see Appendix Table S4. Fluorescent profiles correspond to the mean pixel value of a transversal section of the cell along the mid‐cell axis (see also Kühn *et al*, 2021). For comparison of proteins with different expression levels, the profiles were normalized by the total fluorescence of the cell and rescaled the cell length. Cells were oriented so that the dim pole is at *x* = −1, the bright pole at *x* = 1, and mid‐cell at *x* = 0. Mean profiles and standard deviations were computed for every biological replicate.

### Polar localization index

To quantify the extent of polar localization vs cytoplasmic localization, we computed a polar localization index. We defined a polar area in which we measure the polar fluorescence signals $I_{A}$ and $I_{B}$ of opposite poles A and B according to the ratio between cell width and cell length to account for differences in cell size ($\text{polar area}=\frac{\text{cell width}}{\text{cell length}}*0.5$). Since the fluorescence profiles of purely cytoplasmic proteins are bell‐shaped instead of flat, we applied the following correction method to accurately quantify the polar localization: As baseline profile corresponding to a polar localization index of zero, the fluorescence profile of soluble mNeonGreen expressed from plasmid pJN105‐mNG (uninduced) was determined. We subtracted the average mNG baseline profile plus standard deviation from the measured profiles. The corrected integrated signal at the defined polar areas $I_{\mathrm{Ac}}+I_{\mathrm{Bc}}$ divided by the initial total polar fluorescence $I_{A}+I_{B}$ corresponds to the polar localization index ($\text{polar localization index}=\frac{I_{\mathrm{Ac}}+I_{\mathrm{Bc}}}{I_{A}+I_{B}}$). Values slightly below 0 can occur with this correction for non‐polarly localizing proteins due to noise. In this case, the polar localization index was set to 0. A polar localization index of 0 corresponds to completely cytoplasmic proteins, whereas a value toward 1 corresponds to polarly localized proteins. Due to the applied correction method values of exactly 1 can never be reached, even for proteins that would localize exclusively at the poles. This correction method differs from the method we used previously (Kühn *et al*, 2021) because we found it to be more accurate for proteins clearly localizing to the poles but to a low extent. To judge this, we compared the polar localization indexes from our previous and new analysis with the raw images as well as the fluorescent profiles (which are unaffected by this correction method). Most importantly, the polar localization indexes of proteins with weak but clearly visible polar localization (for example in Fig 6A and B) were slightly above 0 with the new analysis but below zero or zero at all timepoints with the previously used analysis.Therefore, polar localization indexes shown here cannot be directly compared to the indexes published in Kühn *et al* (2021).

### Asymmetry index

We similarly computed an asymmetry index (previously called symmetry index in Kühn *et al*, 2021) by taking the ratio between the maximum total fluorescence $I_{\max}$ of opposite poles A and B and the sum of the polar total fluorescence $I_{A}+I_{B}$ ($\text{asymmetry index}=\frac{I_{\max}}{I_{A}+I_{B}}$). A value of 0.5 corresponds to a perfectly symmetric bipolar localization, whereas a value of 1 corresponds to a perfectly unipolar (asymmetric) localization. Note, we found that even proteins with visually symmetric polar localization or no polar localization at all have asymmetry indexes closer to 0.55 than 0.5. This likely originates from a bias that is due to noise of the fluorescence and orienting the bright cell pole to the right by default.

### Average localization pattern

We generated average localization maps of whole cells using the demograph function of BacStalk. We selected 42 cells with similar length around 4 μm ± 0.2 μm from all segmented cells. We randomly selected one replicate for PilG and PilH that were imaged the same day. The cell lengths and fluorescent intensities were normalized and subsequently averaged using a custom ImageJ script. Note that these average maps only give a rough visual representation of protein localization and do not include all data that were acquired and used to generate quantitative localization indexes as described above.

### Frequency of protein pole‐to‐pole switches

Cells were prepared and fluorescence image sequences recorded essentially as described above. Cells were grown and imaged in a heated chamber (37°C) on the microscope at 0.2 frames per second for 3 min after 10 and 70 min. We did not image cells right after surface contact to let the agarose pad settle down to avoid drift. We measured the fluorescence signal at both poles for each cell track and each frame. We generated a series of numbers (1 or −1) corresponding to a given pole (arbitrary but same over the whole track) being the bright pole (1) or the dim pole (−1) for each frame. A pole‐to‐pole switch of the fluorescently labeled protein was counted every time the sign changed. The displayed rate of pole‐to‐pole switches corresponds to the total detected number of switches divided by the total tracked time over all tracks per replicate.

### Spatiotemporal cumulative displacement maps

Cells were prepared and imaged as described above. The analysis was carried out as described previously (Kühn *et al*, 2021). Movies of single twitching cells were recorded by phase contrast microscopy at 0.2 frames per second for 5 min at room temperature. Movies were processed with a custom ImageJ macro to ensure compatibility with the downstream analysis. Briefly, movies were cut into quadrants, drift was corrected if necessary using the StackReg plugin (version July 7, 2011; Thévenaz *et al*, 1998), and image sequences were saved as individual tif images. BacStalk (version 1.8; Hartmann *et al*, 2020) was used to segment and track cells. Cell tracks were analyzed with a custom MATLAB script. Briefly, we defined a cell orientation unit vector t^→^ from the center of mass (CM) to the initial leading pole. We determined the initial leading pole by comparing the scalar products of the unit vectors from CM to poles A and B (arbitrary classification) to the normalized displacement vector d^→^ in the first frame in which the cell was classified as moving (speed threshold: one pixel per frame for at least three consecutive frames). We determined the cell displacement δ relative to the initial leading pole using vectors t^→^ and d^→^. δ is positive if cells twitch forward and negative if they reverse and twitch backward. We generated spatiotemporal displacement maps by cumulating the direction‐corrected displacement as a function of time.

### Reversal frequency of isolated cells

Cells were prepared and movies recorded as described above. The analysis was carried out as described previously (Kühn *et al*, 2021). Briefly, for each frame (starting from the first frame in which the cell was classified as moving), the scalar product between the normalized displacement vector d^→^ and the cell orientation unit vector t^→^ was determined and rounded. This yielded a series of numbers that correspond to movement of the cell toward the initial leading pole (1), toward the initial lagging pole (−1), or no movement (0) at that timepoint (timepoints with no movement were removed). Cells were considered moving in the same direction (relative to the initial leading pole) as long as the sign remained the same and counted reversing when a change of sign occurred. Reversals were only considered if at least two subsequent frames before and after the reversal had the same sign (to correct for frequent sign changes when a cell was close to non‐moving). To calculate the reversal frequency, we divided the sum of all considered reversals by the total tracked time over all cell tracks for each biological replicate. All displayed datapoints correspond to individually repeated biological replicates.

### Reversal frequency after collisions

Cells were prepared and movies recorded as described above. The analysis was carried out as described previously (Kühn *et al*, 2021). We counted cell–cell collisions and potential subsequent reversals manually using the ImageJ plugin Cell Counter. A collision was only considered if the cell was moving for at least three frames in the same direction prior to the collision, and the collision lasted for at least two frames (frame interval 5 s). Collisions with angles below roughly 20° were not considered. We considered a reversal following a collision only if it occurred within five frames after the collision ended. Freshly divided cells were not considered. We calculated the frequency of reversals following a collision by dividing the sum of all considered reversals by the sum of all considered collisions for each biological replicate. All displayed datapoints correspond to individually repeated biological replicates.

### Fluorescent labelling of pili

Cells were essentially prepared and imaged as described above, with the exception of staining T4P prior to microscopy and increasing the fluorescence excitation power from 20 to 50%. We used a strain with mutated *pilA* (*pilA*_*A86C*_; Koch *et al*, 2021) expressed from its chromosomal native locus. Prior to preparing the microscope slide, we labeled PilA_A86C_ by supplementing the culture medium with 5 μl of the maleimide‐conjugate fluorescent dye Alexa Fluor™ 488 C5 maleimide (Thermo Fischer A10254). Cells were incubated in the dark for 30 min, washed with PBS, and used for fluorescence microscopy. Note that only PilA_A86C_ in the extended pili are labeled during the staining process, and more non‐labeled proteins get produced after the washing. Therefore, not all pili are visible in the recorded movies, and some cells may still twitch without visible pili.

### cAMP quantification using PaQa‐YFP and PlacP1‐YFP reporter

Single‐cell cyclic adenosine monophosphate (cAMP) levels were measured using the PaQa‐YFP/PrpoD‐mKate2 or PlacP1‐YFP/POXB20‐mKate2 reporter system as described previously (Persat *et al*, 2015a). *P. aeruginosa* was transformed with reporter plasmid pUCP18‐PaQa‐YFP/PrpoD‐mKate2 or pUC18‐PlacP1‐YFP/POXB20‐mKate2. PaQa is a native *P. aeruginosa* promoter responsive to cAMP, whereas PrpoD is a constitutive promoter. LacP1 is a synthetic promoter responsive to cAMP, and OXB20 is a strong constitutive promoter (Oxford Genetics Ltd. (UK), Sigma).

For the PaQa measurements, cells were grown overnight in LB‐carbenicillin, diluted to OD_600_ = 0.05 and grown until mid‐exponential phase (OD_600_ = 0.4–0.8). After dilution to OD_600_ = 0.1, liquid samples were imaged, and in parallel 100 μl of the diluted culture was plated on a 1% agarose plate (LB, no antibiotics). After 3 h at 37°C, cells were harvested by adding 1.5 ml LB to the plate and gently shaking. OD_600_ was measured and set to 0.1 for imaging. Images in YFP and mKate2 channels were acquired. Cells were segmented, and mean cellular fluorescence was measured using BacStalk (Hartmann *et al*, 2020). We then used a custom Python script to compute median PaQa‐YFP to mKate2 fluorescent intensity ratios.

For the LacP1 measurements, strains containing the reporter plasmid were grown on LB agar plates with 400 μg/ml carbenicillin (GoldBio) at 37°C to obtain single colonies. Single colonies were grown in liquid broth in a deep well plate overnight, 37°C, shaking. Overnight cultures were diluted and grown at 37°C for 3 h. After 3 h, a fraction of the bacteria was fixed by the addition of 4% methanol‐free paraformaldehyde (PFA, Thermo Scientific) for 10 min at room temperature, and the reaction was quenched by the addition of 0.3 M glycine. A second fraction of bacteria was spotted onto LB/Carbenicilin plates and grown for 2 h, after 2 h the spots were scraped into PBS and fixed by the addition of PFA and glycine, as above. Samples were diluted in PBS and analyzed on an LSRII flow cytometer the following day in the UCSF Parnassus Flow CoLab, (RRID:SCR_018206). Data were exported from FlowJo and analyzed using the same custom Python script as above. We reduced the sample size from about 50,000 to 1,000 cells per replicate by randomizing and reducing the data from each sample separately.

All displayed datapoints correspond to individual cells from at least three (up to six) individually repeated biological replicates.

### Phos‐tag™ SDSPAGE and immunoblotting to detect phosphorylated PilG *in vivo*

*Pseudomonas aeruginosa* PAO1 and selected mutant strains expressing chromosomal 3xFlag‐PilG were grown on LB agar plates at 37°C to obtain single colonies. Single colonies were grown in liquid LB broth overnight. Overnight cultures were diluted in LB broth and grown at 37°C for 3 h. After 3 h, a fraction of the bacteria was harvested by centrifugation (10,000 *g*, 5 min, 4°C), the pellet was suspended in Laemmli sample buffer (Biorad) with 2.5% 2‐mercaptoethanol and 0.1 U/μl benzonase (Millipore) and frozen at −20°C. A second fraction was plated on LB plates and incubated at 37°C. After 2 h, bacteria were harvested into 1 ml cold buffer (5 mM MgCl_2_ / 50 mM KCl / 50 mM Tris pH 7.5 / 2 mM DTT) and centrifuged at 10,000 *g* for 5 min at 4°C. Supernatants were removed and pellets suspended in cold reducing Laemmli sample buffer (Biorad) containing 0.1 U/μl Benzonase (Millipore) and frozen at −20°C. Protein concentration was measured using Pierce™ 660 nm Protein Assay Reagent with Ionic Detergent Compatibility Reagent. Two μg of each sample was separated by 12% SDS‐PAGE with 100 μM Phos‐tag™ acrylamide (Wako Chemicals, Richmond, VA, USA) and 200 μM MnCl_2_. Phos‐tag™ acrylamide gels were electrophoresed at a constant current of 30 mA in running buffer (25 mM Tris, 192 mM glycine, 0.1% SDS, pH 8.3). Gels were incubated in transfer buffer (25 mM Tris, 192 mM glycine, pH 8.3, 20% methanol) containing 1 mM EDTA for 10 min, followed by incubation in transfer buffer for an additional 20 min. Gels were then transferred (100 V, 60 m) to PVDF membranes, blocked in TBS (20 mM Tris, 150 mM NaCl, pH 7.5)/5% milk, and probed with monoclonal mouse anti‐Flag M2 antibody (affinity isolated, catalog number F3165, Sigma) in TBST (20 mM Tris, 150 mM NaCl, 0.05% Tween20, pH 7.5)/5% milk. Membranes were washed in TBST and incubated with IRDye® 680RD goat anti‐mouse IgG secondary antibody (RRID AB_10956588, catalog number 926‐68070, LI‐COR) and imaged on an LI‐COR Odyssey system. Intensity of individual bands was generated using ImageStudioLite or EmpiriaStudio (LI‐COR). Fraction PilG‐P was calculated from the intensity of the PilG‐P band divided by the total intensity of PilG bands (sum of both PilG bands). All displayed datapoints correspond to individually repeated biological replicates.

### Statistical tests

To test significance, one‐way ANOVA and Tukey's *post hoc* tests were done where required using Python (version 3.8.5). Different groups values were considered significantly different with a *P*‐value below 0.05.

## Author contributions

**Marco J Kühn:** Conceptualization; resources; data curation; software; formal analysis; funding acquisition; validation; investigation; visualization; methodology; writing – original draft; project administration; writing – review and editing. **Henriette Macmillan:** Conceptualization; resources; data curation; formal analysis; validation; investigation; visualization; methodology; project administration; writing – review and editing. **Lorenzo Talà:** Conceptualization; resources; software; formal analysis; visualization; methodology. **Yuki Inclan:** Conceptualization; resources; data curation; formal analysis; validation; investigation; visualization; methodology; project administration; writing – review and editing. **Ramiro Patino:** Conceptualization; resources; data curation; formal analysis; validation; investigation; methodology; project administration. **Xavier Pierrat:** Resources. **Zainebe Al‐Mayyah:** Resources. **Joanne N Engel:** Conceptualization; supervision; funding acquisition; methodology; project administration; writing – review and editing. **Alexandre Persat:** Conceptualization; data curation; supervision; funding acquisition; methodology; writing – original draft; project administration; writing – review and editing.

## Disclosure and competing interests statement

The authors declare that they have no conflict of interest.

## Supporting information



Appendix

Movie EV1

Movie EV2

Movie EV3

Movie EV4

## Data Availability

All figure source data can be found in BioStudies under the accession number S‐BSST1007 (https://www.ebi.ac.uk/biostudies/studies/S‐BSST1007). Custom ImageJ, Python, and MATLAB codes are available on Github (https://github.com/PersatLab/antagonists). Raw images are available from the corresponding authors upon request.
