# Supplementary material for: Two antagonistic response regulators control Pseudomonas aeruginosa polarization during mechanotaxis
Source: EMBO J. 2023 Feb 16;42(7):e112165. doi: 10.15252/embj.2022112165 (PMC10519157; doi:10.15252/embj.2022112165)
Supplement: Supplementary file 1 — Appendix [file EMBJ-42-e112165-s002.pdf]

Appendix

Two antagonistic response regulators control *Pseudomonas aeruginosa* polarization during mechanotaxis.

Authors

Marco J. Kühn[1], Henriette Macmillan[2], Lorenzo Talà[1], Yuki Inclan[2], Ramiro Patino[2], Xavier Pierrat[1], Zainebe Al-Mayyah[1], Joanne N. Engel*[,2,3] and Alexandre Persat*[,1]

Affiliations

[1]Institute of Bioengineering and Global Health Institute, School of Life Sciences, EPFL, Lausanne, Switzerland

[2]Department of Medicine, University of California, San Francisco, USA

[3]Department of Microbiology and Immunology, University of California, San Francisco, USA

*Correspondence to: alexandre.persat@epfl.ch, joanne.engel@ucsf.edu

Content

- Appendix Figures S1-17
- Appendix Tables S1-4
- Appendix References

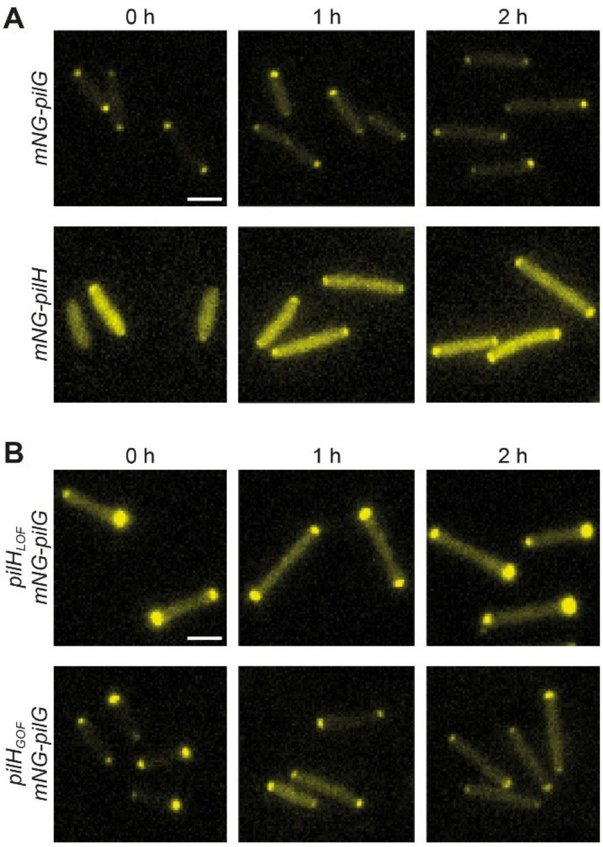

Appendix Figure S1: Example fluorescence microscopy images of mNG-PilG and mNG-PilH in cells grown on a surface. Cells were transferred from liquid culture to solid substrates and imaged immediately (0h) and after 1h and 2h in which the cells adapt to a surface-associated lifestyle. (A) Images correspond to data shown in Figure 1. (B) Images correspond to data shown in Figure 7. Scale bars, 2 µm.

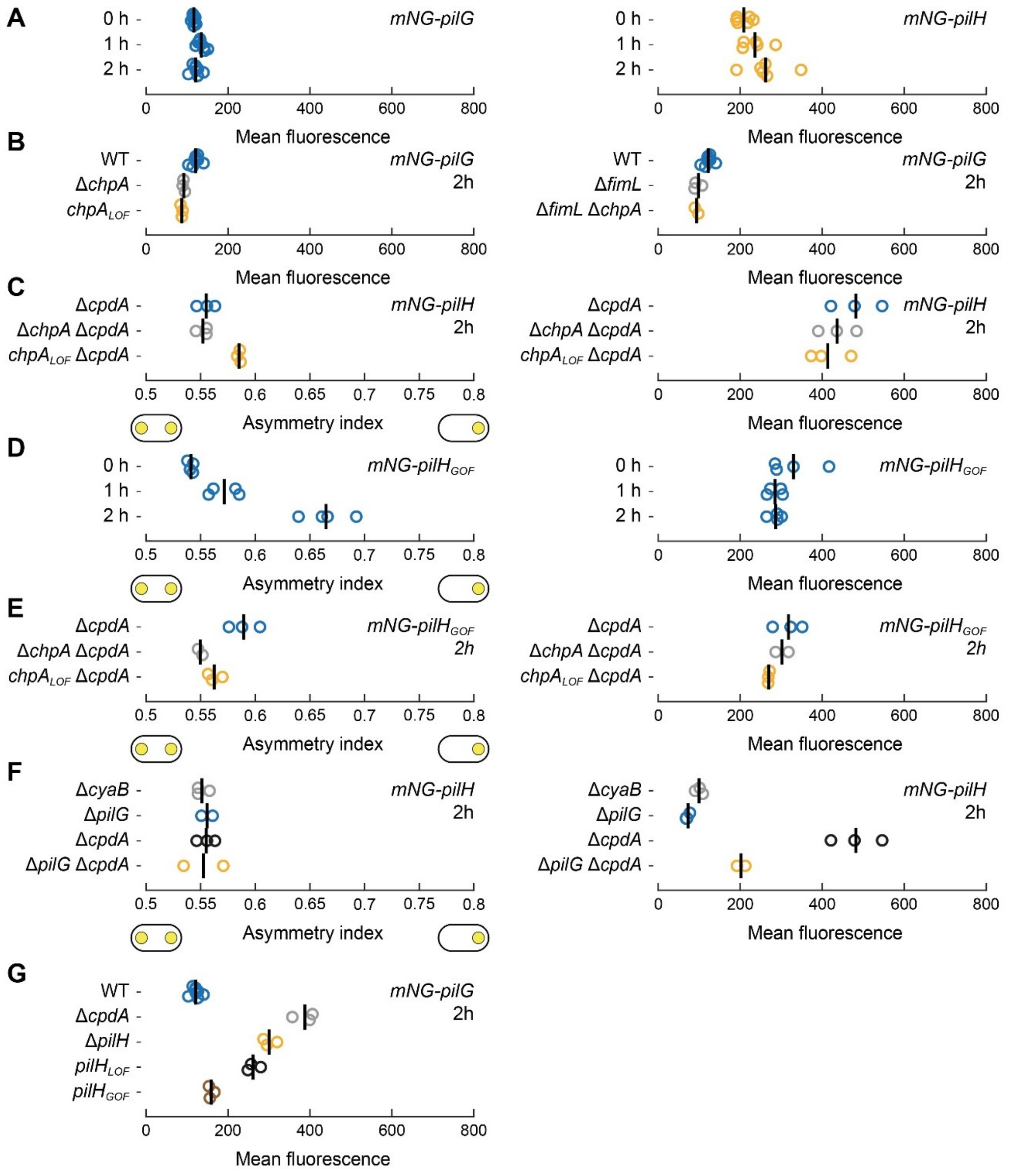

Appendix Figure S2: Mean cell fluorescence and asymmetry indexes of mNG-PilG and mNG-PilH. Panels correspond to main figures as follows: (A) Figure 1 (B) Figure 2 (C) Figure 3 (D) Figure 4AB (E) Figure 4CD (F) Figure 6 (G) Figure 7. Note, for panels C and E, *cpdA* was deleted in all display strains to rescue cAMP levels and mitigate the negative effects caused by low cAMP. Circles, median of each biological replicate. Vertical bars: mean across biological replicates.

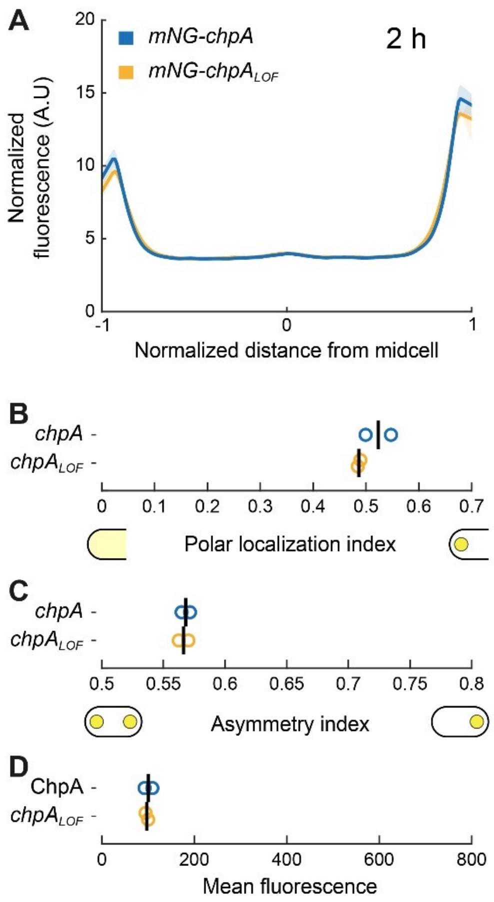

Appendix Figure S3: ChpA and ChpA$_{LOF}$ localize similarly to the poles. (A) Average fluorescence profiles of mNG-ChpA after 2h surface growth. Note, wild-type mNG-tagged ChpA is non-functional (0 moving cells found in 97 tracked cells) like the loss-of-function mutant *chpA$_{LOF}$* (2 moving cells found in 254 tracked cells). (B) Quantification of polar localization, (C) asymmetry index and (D) mean cellular fluorescence. Solid lines, mean normalized fluorescence profiles across biological replicates. Shaded area, standard deviation across biological replicates. Circles, median of each biological replicate. Vertical bars, mean across biological replicates.

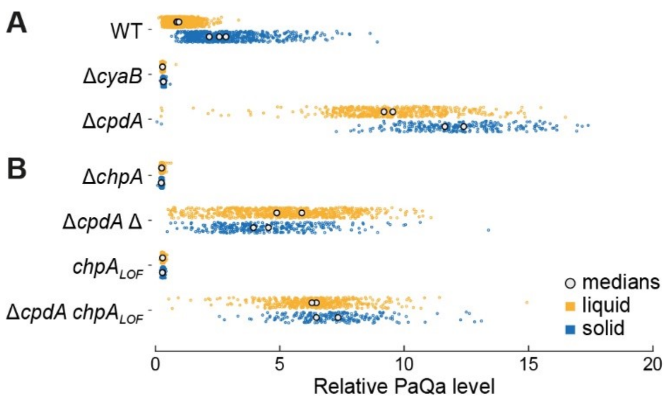

Appendix Figure S4: Quantification of cAMP levels of *chpA* mutants measured by PaQa-YFP reporter fluorescence. (A) Relative cAMP levels measured by PaQa reporter fluorescence in deletion mutants of the cAMP production cycle. Δ*cyaB* results in constitutively low and Δ*cpdA* in constitutively high cAMP levels. The cAMP level of Δ*cpdA* increases on surfaces, likely due to increased activity of the adenylate cyclase CyaB (Fulcher *et al*, 2010). (B) Relative cAMP levels of Δ*chpA*, the histidine kinase of the Chp system, and $chpA_{LOF}$. Note, deletion of *cpdA* partially rescues the low cAMP level of *chpA* mutants, however, cAMP levels don't reach the high cAMP levels of Δ*cpdA*. Very likely, CyaB doesn't get activated in *chpA cpdA* double mutants (Fulcher *et al*, 2010). Therefore, cAMP levels don't increase upon surface contact. Colored circles represent measurements of single cells, white circles correspond to medians of biological replicates. All displayed strains are in Δ*fliC* background.

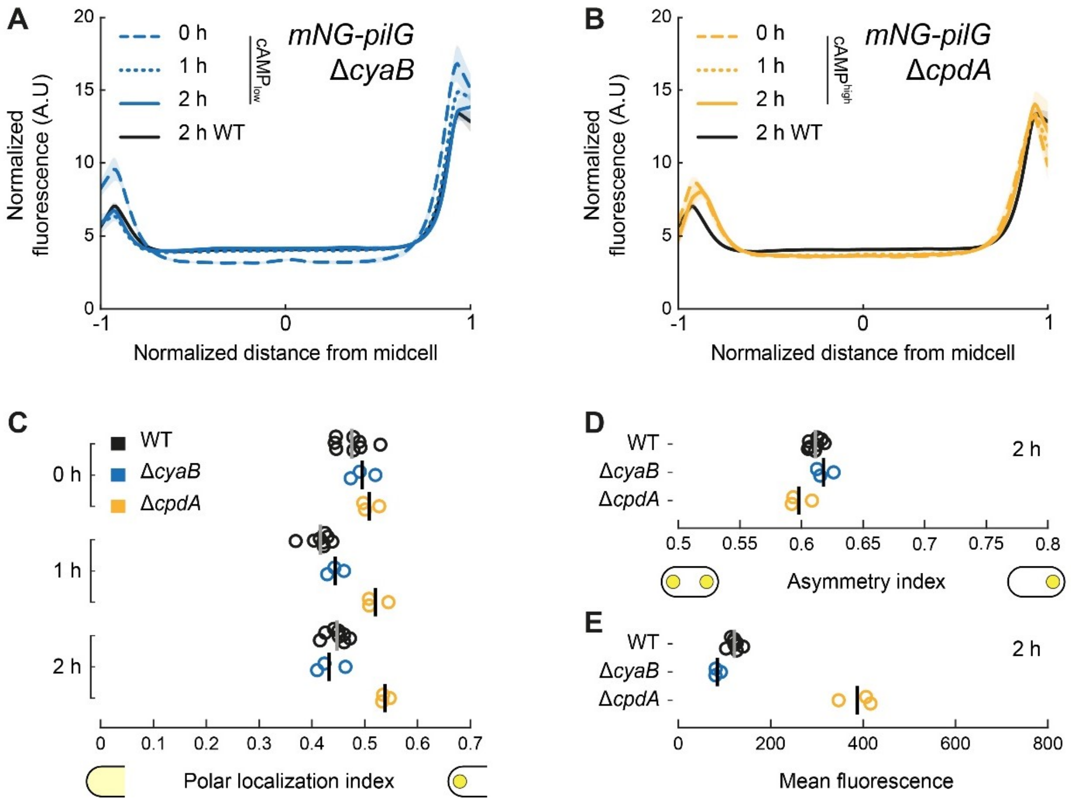

Appendix Figure S1: Time course of cAMP-dependent localization of mNG-PilG in cells grown on solid substrate. Average fluorescence profiles of mNG-PilG in (A) low and (B) high cAMP. Quantification of (C) polar localization, (D) asymmetry index and (E) mean cellular fluorescence. In low cAMP (Δ*cyaB*), the localization pattern as well as the decrease of polar localization over time is almost identical to WT. Data from Figure 1D included for comparison (black). Note, WT refers to a strain without *cyaB* or *cpdA* deletion. In high cAMP (Δ*cpdA*), polar localization of PilG is comparable to WT at 0h; however, it remains high over time. The higher polar localization is due to increased localization to the dim pole (at x = -1, panel B) which is reflected by a slightly lower asymmetry index (D), i.e. more symmetric localization. The fluorescent signal of mNG-PilG, as a proxy for protein concentration, is largely unaffected by low cAMP; however, it is strongly increased in high cAMP. Solid lines, mean normalized fluorescence profiles across biological replicates. Shaded area, standard deviation across biological replicates. Circles, median of each biological replicate. Vertical bars, mean across biological replicates.

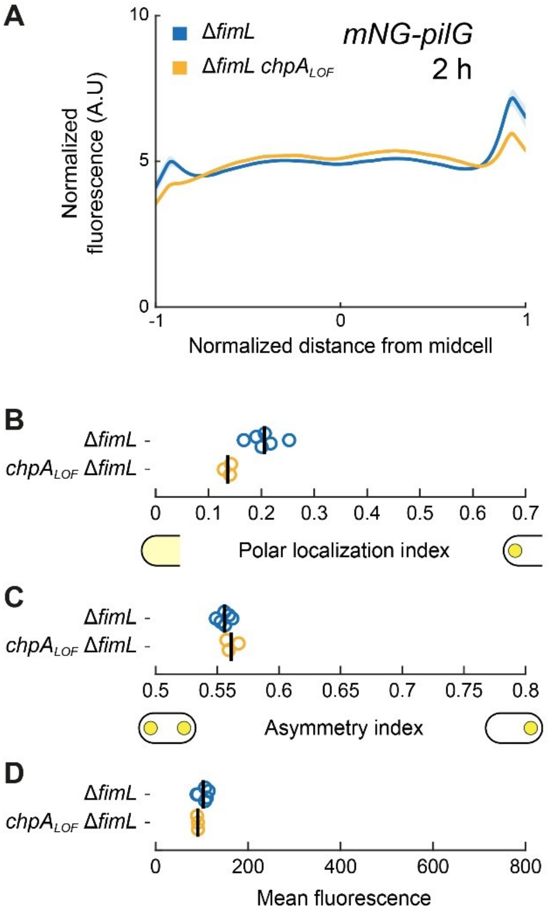

Appendix Figure S6: ChpA plays a role in recruiting PilG independently of phosphorylation. (A) Average fluorescence profiles of mNG-PilG after 2h surface growth. In Δ*fimL*, PilG is recruited to the poles only by ChpA. Without the ability to phosphorylate, $ChpA_{LOF}$ still recruits PilG, although to a lesser extent than wild-type ChpA. (B) Quantification of polar localization, (C) asymmetry index and (D) mean cellular fluorescence. Solid lines, mean normalized fluorescence profiles across biological replicates. Shaded area, standard deviation across biological replicates. Circles, median of each biological replicate. Vertical bars, mean across biological replicates.

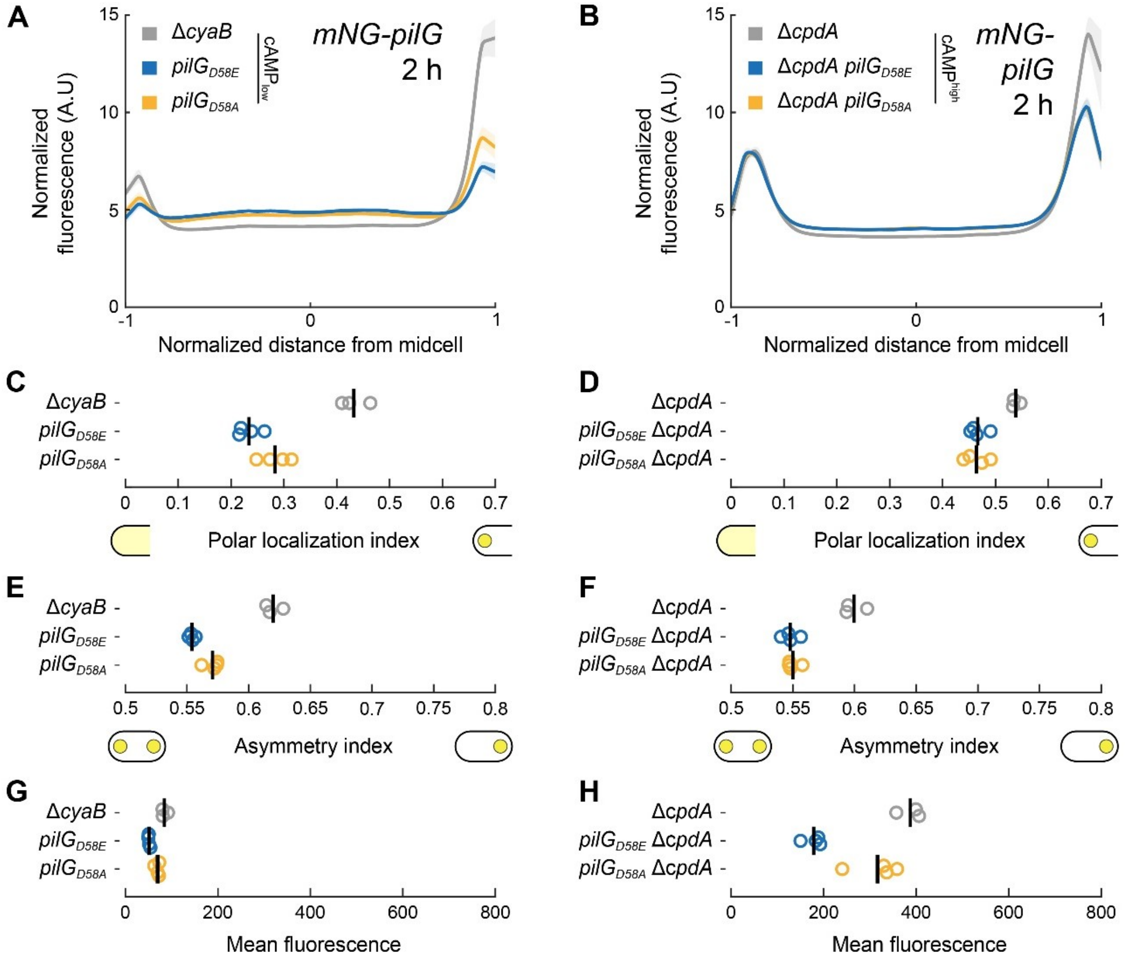

Appendix Figure S7: PilG$_{D58A}$ and PilG$_{D58E}$ similarly localize to the poles. Average fluorescence profiles of mNG-PilG point mutants after 2h surface growth in (A) low and (B) high cAMP. (C,D) Quantification of polar localization, (E,F) asymmetry index and (G,H) mean cellular fluorescence. Polar localization and asymmetry indexes of PilG$_{D58A}$ and PilG$_{D58E}$ are decreased compared to WT in low cAMP. Polar localization differs only slightly between the mutants, and the differences disappear in high-cAMP. Solid lines, mean normalized fluorescence profiles across biological replicates. Shaded area, standard deviation across biological replicates. Circles, median of each biological replicate. Vertical bars, mean across biological replicates.

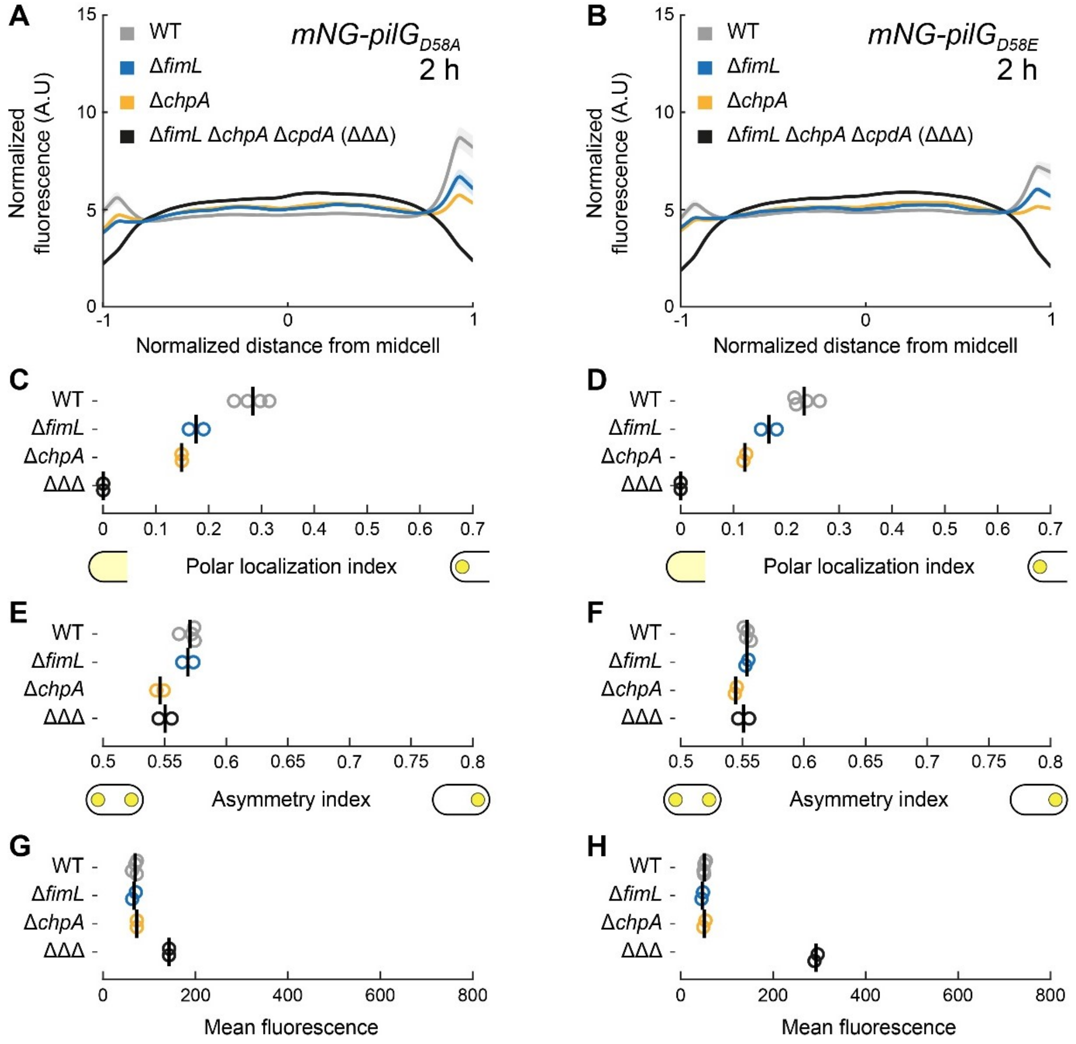

Appendix Figure S8: D58A and D58E mutations of PilG do not interfere with FimL- and ChpA-dependent recruitment. Average fluorescence profiles of (A) mNG-$PilG_{D58A}$ and mNG-$PilG_{D58E}$ (B) after 2h surface growth. Polar localization of both point mutants similarly depends on FimL and ChpA, comparable to wild-type PilG. (C,D) Quantification of polar localization, (E,F) asymmetry index and (G,H) mean cellular fluorescence of $PilG_{D58A}$ and mNG-$PilG_{D58E}$, respectively. ΔΔΔ refers to strain Δ*fimL* Δ*chpA* Δ*cpdA*. Solid lines, mean normalized fluorescence profiles across biological replicates. Shaded area, standard deviation across biological replicates. Circles, median of each biological replicate. Vertical bars, mean across biological replicates.

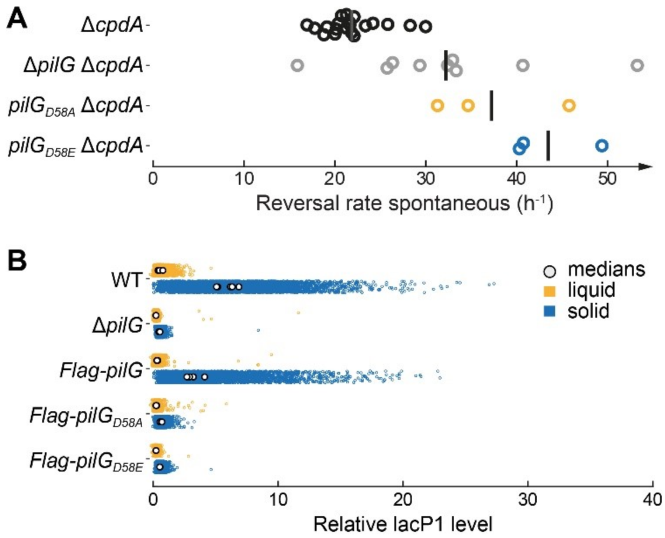

Appendix Figure S9: Reversal rates and cAMP production of *pilG*$_{D58}$ point mutants. (A) Spontaneous reversal rates of *pilG*$_{D58A}$ and *pilG*$_{D58E}$ mutants. To bypass low cAMP levels *cpdA* was deleted in all displayed strains. Both *pilG* mutants show increased reversals rates similar to Δ*pilG* Indicating a loss of function. (B) Quantification of cAMP levels measured by PlacP1-YFP reporter fluorescence. Low cAMP levels of both *pilG* mutants confirm that both point mutations result in a loss of function. The Flag tag alone does not confound the cAMP measurements. Colored circles represent measurements of single cells, white circles correspond to medians of biological replicates.

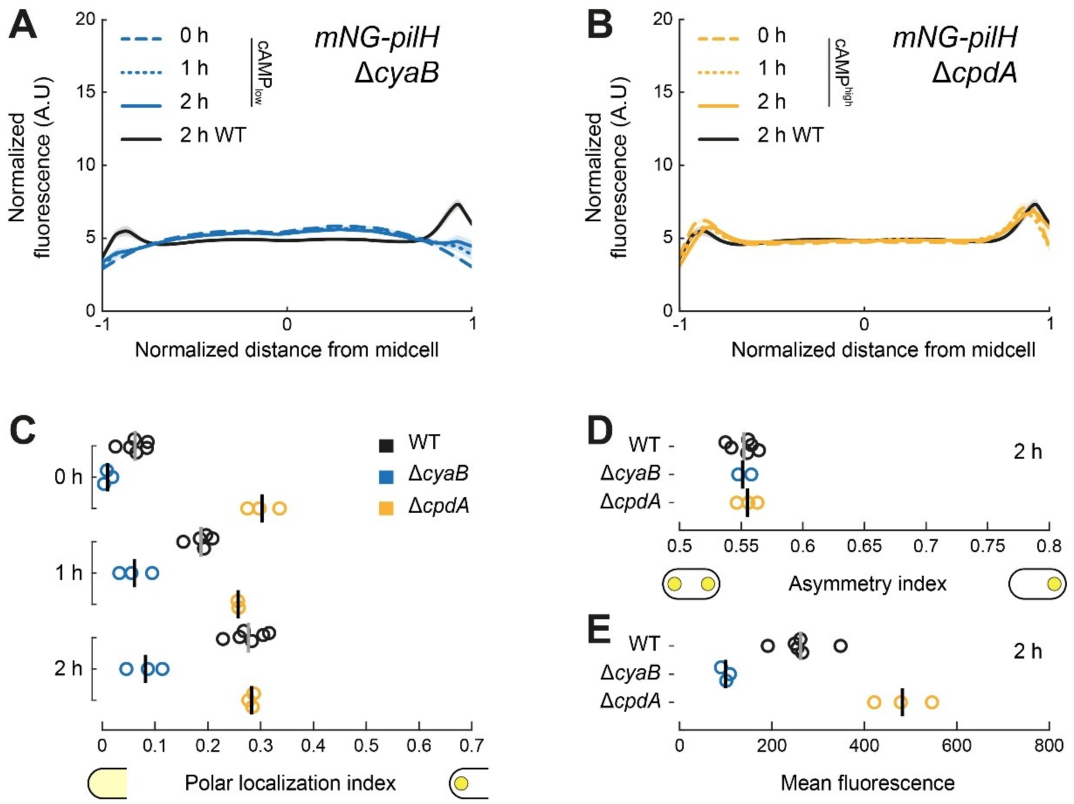

Appendix Figure S10: Time course of cAMP-dependent localization of mNG-PilH in cells grown on solid substrate. Average fluorescence profiles of mNG-PilH in (A) low and (B) high cAMP, respectively. Quantification of (C) polar localization, (D) asymmetry index and (E) mean cellular fluorescence. In low cAMP (Δ*cyaB*), polar localization is generally reduced, however, recruitment of PilH to the poles over time still takes place. In high cAMP (Δ*cpdA*), polar localization is always as high as in WT after 2h surface growth. Data from Figure 1 included for comparison (black). Note, WT refers to a strain without *cyaB* or *cpdA* deletion. The fluorescent signal of mNG-PilH, as a proxy for protein concentration, is proportional to the cAMP level in *cyaB* and *cpdA* mutants (cf. Appendix Figure S4A). Solid lines, mean normalized fluorescence profiles across biological replicates. Shaded area, standard deviation across biological replicates. Circles, median of each biological replicate. Vertical bars, mean across biological replicates.

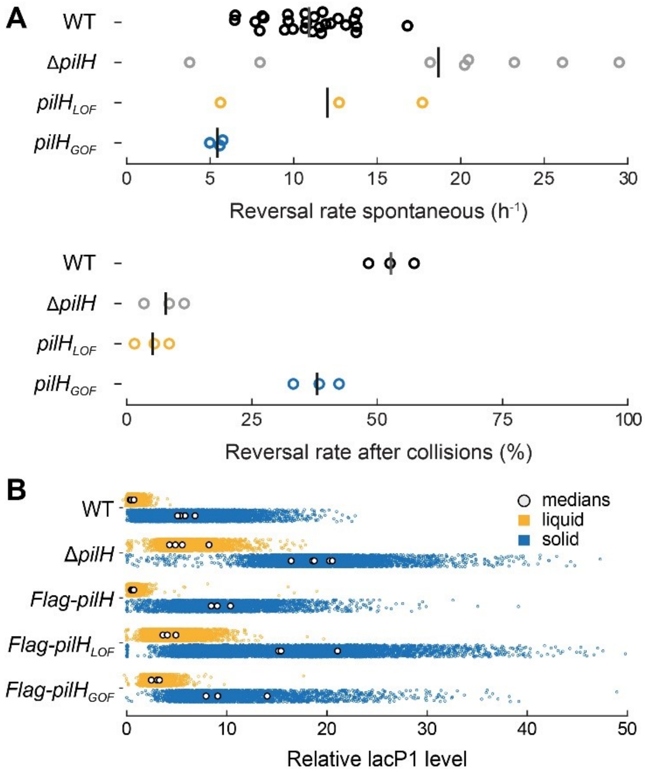

Appendix Figure S11: Reversal rates and cAMP production of loss- and gain-of-function mutants of PilH. (A) Spontaneous reversal rates and reversal rates after collision of *pilH*$_{LOF}$ and *pilH*$_{GOF}$ mutants. Δ*pilH* and *pilH*$_{LOF}$ show low to almost no reversals (see also (Kühn *et al*, 2021)). *pilH*$_{GOF}$ rescues reversal rates, however, doesn't fully restore wild-type rates. Because of high pili number in mutants with high cAMP level resulting in erratic movement of cells, spontaneous reversals rates are not measured very precisely by our algorithm. Manually counted reversal rates after collision give a better estimate. Δ*pilH* collision data from (Kühn *et al*, 2021). (B) Quantification of cAMP levels measured by PlacP1-YFP reporter fluorescence. cAMP levels of *pilH*$_{LOF}$ are comparable to Δ*pilH*, whereas *pilH*$_{GOF}$ partially rescues cAMP to intermediate levels between WT and Δ*pilH* or *pilH*$_{LOF}$. Colored circles represent measurements of single cells, white circles correspond to medians of biological replicates.

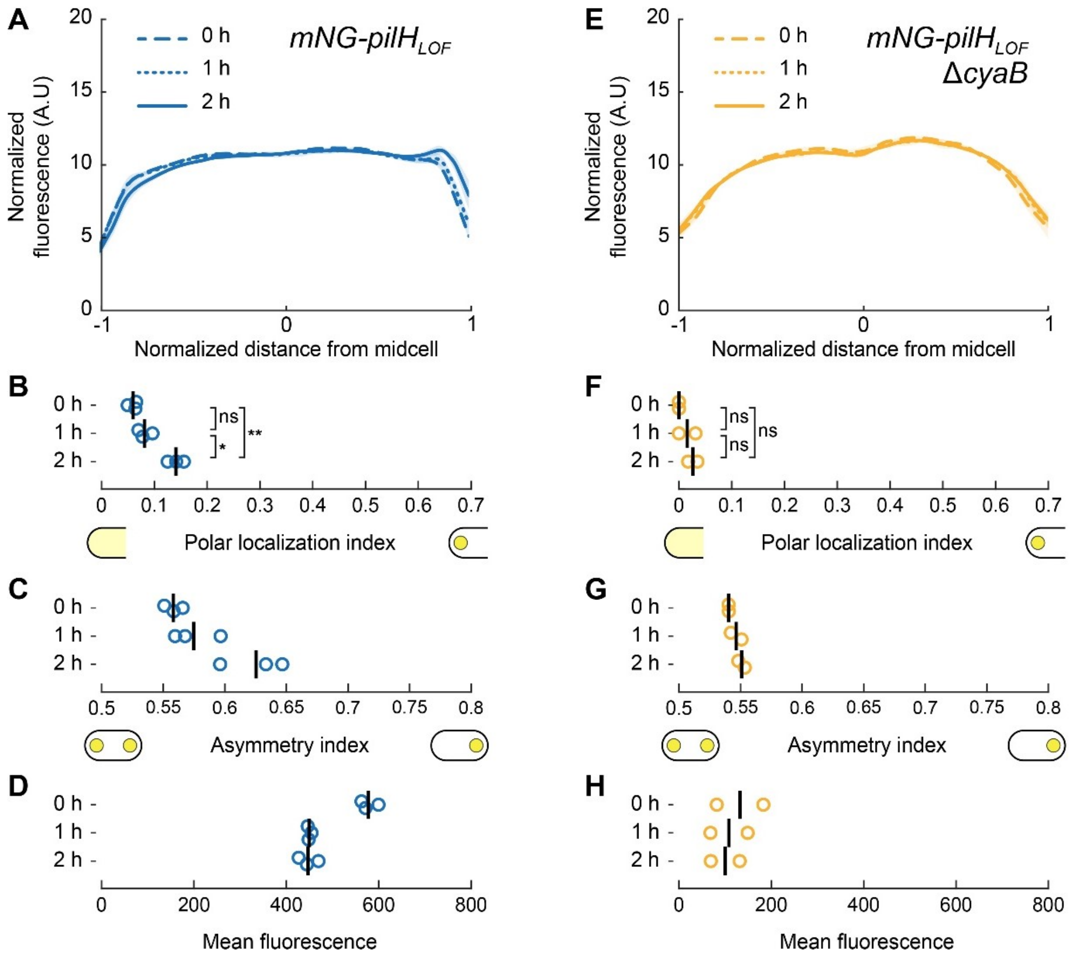

Appendix Figure S12: PilH locked in its inactive conformation is recruited to the poles upon surface contact. (A) Time course of localization profiles of mNG-PilH_{LOF} in cells grown on solid substrate. Quantification of corresponding (B) polar localization, (C) asymmetry index and (D) mean cellular fluorescence. Like PilH_{wt} and PilH_{GOF}, PilH_{LOF} gets recruited to the poles over time (B). However, the effect is significantly less pronounced, possibly due to saturation effects because of high fluorescent signal. Note, loss-of-function mutation of *pilH* results in high cAMP like in Δ*pilH* (Appendix Figure S11). (E-H) Same analysis in low cAMP (Δ*cyaB*). Polar recruitment may take place but the effect is too weak to be measured clearly (F). Solid lines, mean normalized fluorescence profiles across biological replicates. Shaded area, standard deviation across biological replicates. Circles, median of each biological replicate. Vertical bars, mean across biological replicates. *, $p<0.05$; **, $p\leq0.001$; ns, not significant.

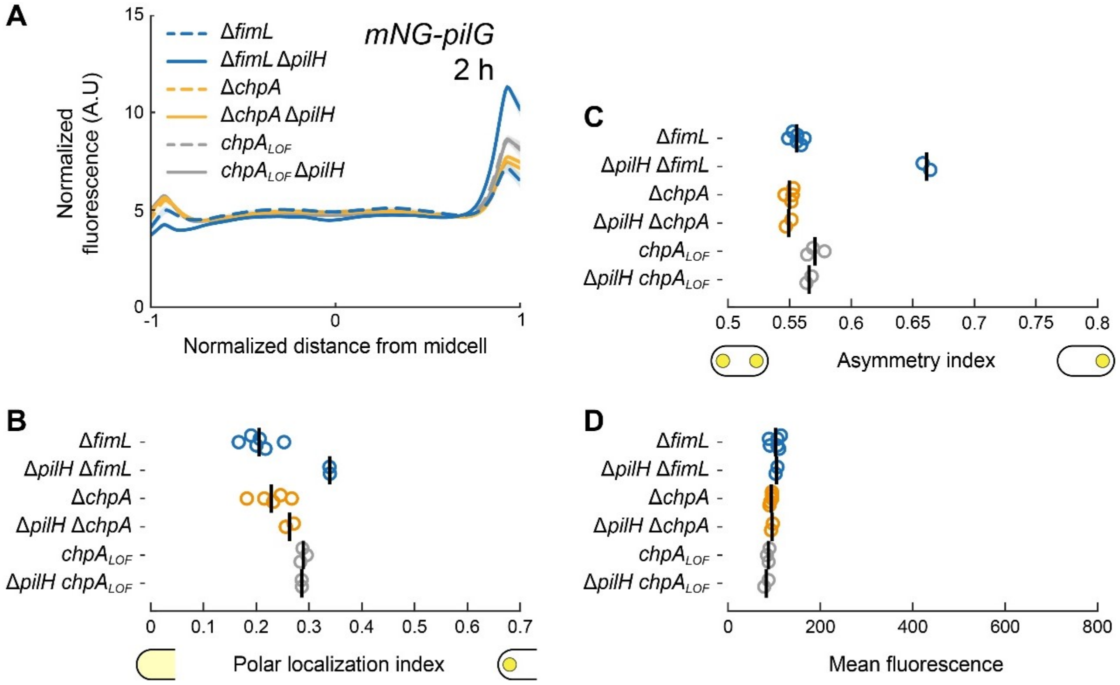

Appendix Figure S13: PilH requires functional ChpA but not FimL to modulate PilG polar localization. (A) Average fluorescence profiles of mNG-PilG after 2h surface growth. (B) Quantification of polar localization, (C) asymmetry index and (D) mean cellular fluorescence. PilH can repress PilG polar localization only in cells with functional ChpA (only in Δ*fimL*). Solid lines, mean normalized fluorescence profiles across biological replicates. Shaded area, standard deviation across biological replicates. Circles, median of each biological replicate. Vertical bars, mean across biological replicates.

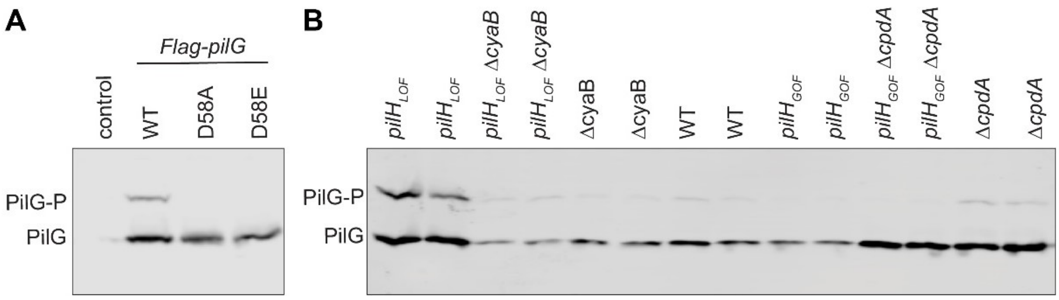

Appendix Figure S14: Example PhosTag™ western blots. (A) Representative gel of PilG and corresponding point mutants that can't get phosphorylated. A slower migrating band corresponding to PilG-P is detected in whole cell lysates from wild type cells expressing Flag-PilG but not in cells expressing PilG with mutations in the phospho-accepting site D58. Control, wild-type PilG without Flag tag. (B) Representative gel of PilG in PilH point mutants and mutants with altered cAMP levels (Δ*cpdA*, Δ*cyaB*), corresponding to data shown in Figure 7GH.

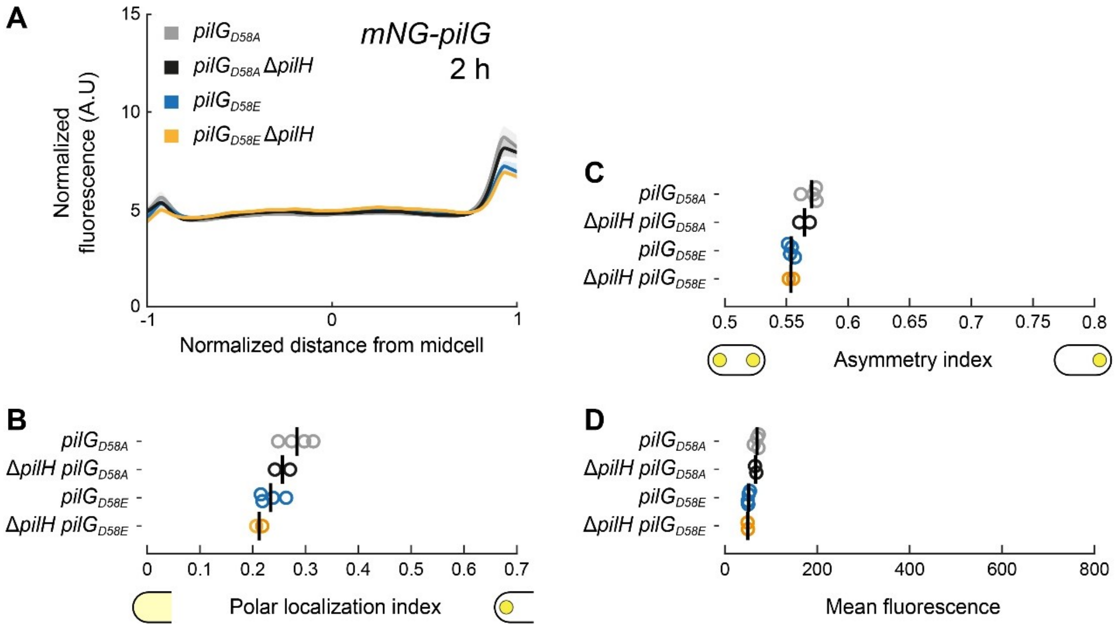

Appendix Figure S15: PilH has no effect on polar localization of non-phosphorylatable PilG mutants. (A) Average fluorescence profiles of mNG-PilH point mutants after 2h surface growth. (B) Quantification of polar localization, (C) asymmetry index and (D) mean cellular fluorescence. Solid lines, mean normalized fluorescence profiles across biological replicates. Shaded area, standard deviation across biological replicates. Circles, median of each biological replicate. Vertical bars, mean across biological replicates.

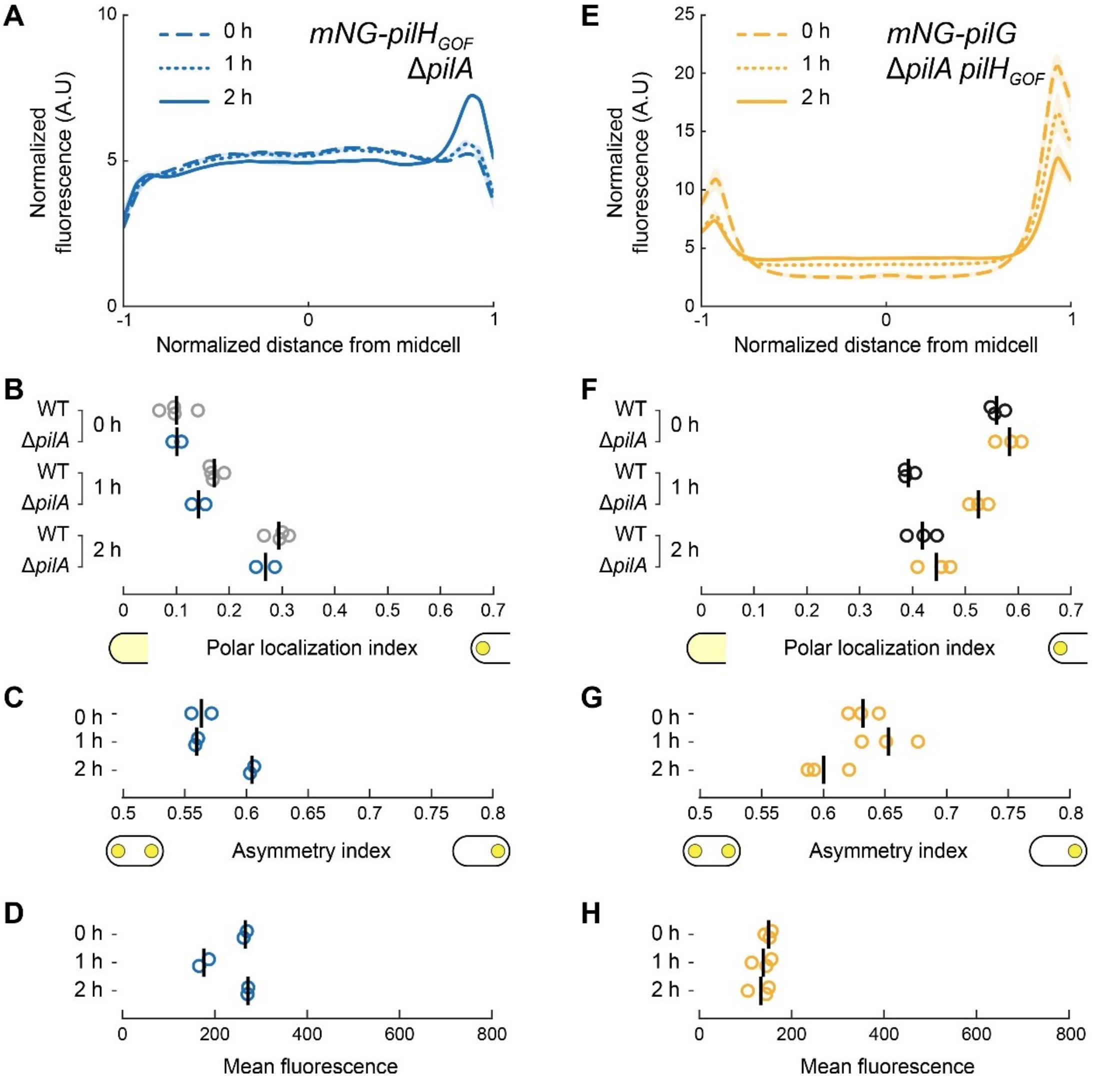

Appendix Figure S16: PilH$_{GOF}$ localization and PilH$_{GOF}$-dependent regulation of PilG in Δ*pilA*. Time course of localization profiles of (A) mNG-PilH$_{GOF}$ in Δ*pilA* and (E) mNG-PilG in Δ*pilA pilH$_{GOF}$* in cells grown on solid substrate. Quantification of corresponding (B,) polar localization, (C) asymmetry index and (D) mean cellular fluorescence. While the overall localization is similar between WT and Δ*pilA*, the change over time is delayed in Δ*pilA* compared to WT. Solid lines, mean normalized fluorescence profiles across biological replicates. Shaded area, standard deviation across biological replicates. Circles, median of each biological replicate. Vertical bars, mean across biological replicates.

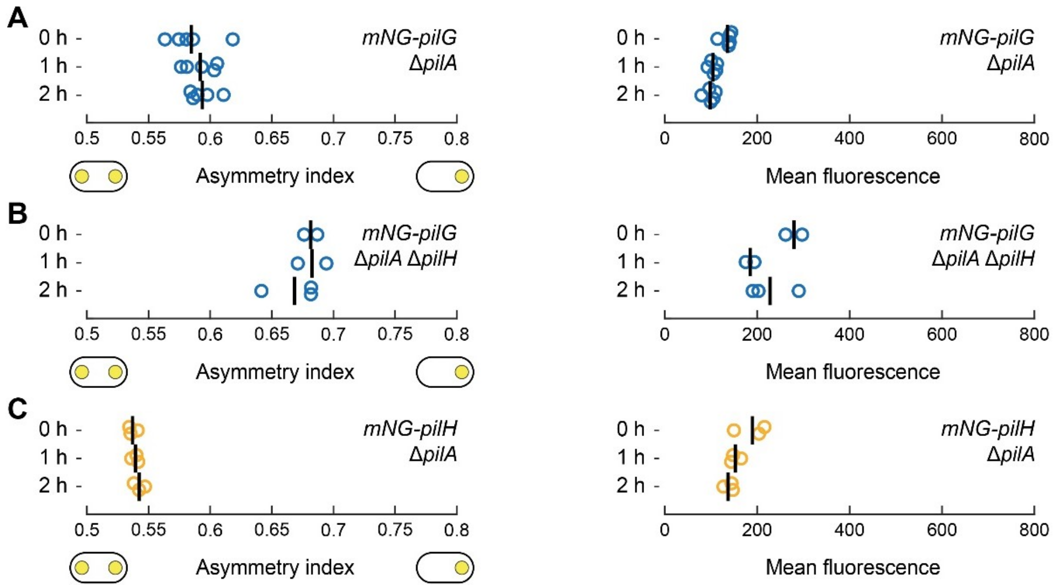

Appendix Figure S17: Asymmetry indexes and mean cell fluorescence. Data for (A) *mNG-pilG ΔpilA*, (B) *mNG-pilG ΔpilA ΔpilH*, (C) *mNG-pilH ΔpilA* corresponding to Figure 8. Circles, median of each biological replicate. Vertical bars: mean across biological replicates.

Appendix Table S1: Strains used in this study.

Name and relevant genotype	Source / Reference	Identifier
Pseudomonas aeruginosa PAO1	(Holloway & Morgan, 1986)	ATCC 15692
Escherichia coli DH5α (hsdR rec lacZYA ϕ80 lacZM15)	Invitrogen	Na
Escherichia coli strain S17.1 (thi pro hsdR recA RP4-2(Tc::Mu)(Km::Tn7))	Stratagene	Na
PAO1 Δ*fliC* (in-frame deletion of PA1092)	(Bertrand *et al*, 2010)	177
PAO1 Δ*pilG* (in-frame deletion of PA0408)	(Bertrand *et al*, 2010)	226
PAO1 Δ*pilH* (in-frame deletion of PA0409)	(Barken *et al*, 2008)	178
PAO1 Δ*chpA* (in-frame deletion PA0413)	(Holloway & Morgan, 1986)	170
PAO1 Δ*cyaB* (in-frame deletion of PA3217)	(Inclan *et al*, 2011b)	174
PAO1 Δ*cpdA* (in-frame deletion of PA4969)	(Inclan *et al*, 2011b)	180
PAO1 Δ*fimL* (in-frame deletion of PA1822)	(Whitchurch *et al*, 2005)	179
PAO1 Δ*fliC* Δ*cyaB*	(Kühn *et al*, 2021)	326
PAO1 Δ*fliC* Δ*cpdA*	(Kühn *et al*, 2021)	337
PAO1 Δ*fliC* Δ*pilG*	(Kühn *et al*, 2021)	330
PAO1 Δ*fliC* Δ*pilH*	(Kühn *et al*, 2021)	232
PAO1 Δ*fliC* Δ*pilG* Δ*cpdA*	(Kühn *et al*, 2021)	459
PAO1 Δ*fliC* Δ*pilG* Δ*pilH*	this study	553
PAO1 Δ*fliC* Δ*pilG* Δ*pilH* Δ*cpdA*	this study	1278
PAO1 Δ*fliC* Δ*chpA* Δ*cpdA*	this study	342
PAO1 Δ*fliC* $chpA_{\Delta HK}$ (deletion of the histidine kinase domain, residues 1943-2176, parent for $chpA_{LOF}$)	this study	1511
PAO1 Δ*fliC* ΔcpdA $chpA_{\Delta HK}$	this study	1512
PAO1 Δ*fliC* $chpA_{LOF}$ (loss-of-function mutations D2086A, D2087A, G2088A)	This study, based on (Bertrand *et al*, 2010)	1536
PAO1 Δ*fliC* Δ*cpdA* $chpA_{LOF}$	this study	1537

PAO1 Δ*fliC pilG*$_{D58A}$ Δ*cpdA* (loss-of-function mutation D52A)	this study	1211
PAO1 Δ*fliC pilG*$_{D58E}$ Δ*cpdA* (loss-of-function mutation D58E)	this study	1171
PAO1 Δ*fliC pilH*$_{LOF}$ (loss-of-function mutation D52A)	this study	1172
PAO1 Δ*fliC pilH*$_{LOF}$ Δ*cyaB*	this study	1166
PAO1 Δ*fliC pilH*$_{GOF}$ (gain-of-function mutation D52E)	this study	1155
PAO1 Δ*fliC pilH*$_{GOF}$ Δ*cyaB*	this study	1167
PAO1 Δ*fliC mNG-pilG* (N-terminal fluorescent fusion to mNeonGreen, GGGGG linker, native locus)	(Kühn *et al*, 2021)	923
PAO1 Δ*fliC mNG-pilG* Δ*cyaB*	this study	1098
PAO1 Δ*fliC mNG-pilG* Δ*cpdA*	this study	1429
PAO1 Δ*fliC mNG-pilH* (N-terminal fluorescent fusion to mNeonGreen, GGGGG linker, native locus)	(Kühn *et al*, 2021)	315
PAO1 Δ*fliC mNG-pilH* Δ*cyaB*	this study	1095
PAO1 Δ*fliC mNG-pilH* Δ*cpdA*	this study	1055
PAO1 Δ*fliC mNG-chpA* (N-terminal fluorescent fusion to mNeonGreen, GGGGG linker, native locus)	this study	312
PAO1 Δ*fliC* mNG-*chpA*$_{\Delta HK}$	this study	1510
PAO1 Δ*fliC* mNG-*chpA*$_{LOF}$	this study	1535
PAO1 Δ*fliC mNG-pilG* Δ*chpA*	this study	1423
PAO1 Δ*fliC mNG-pilG* Δ*chpA* Δ*cpdA*	this study	1464
PAO1 Δ*fliC mNG-pilG chpA*$_{\Delta HK}$	this study	1517
PAO1 Δ*fliC mNG-pilG chpA*$_{LOF}$	this study	1542
PAO1 Δ*fliC mNG-pilG chpA*$_{\Delta HK}$ Δ*cpdA*	this study	1518
PAO1 Δ*fliC mNG-pilG chpA*$_{LOF}$ Δ*cpdA*	this study	1578
PAO1 Δ*fliC mNG-pilG* Δ*fimL*	this study	1045
PAO1 Δ*fliC mNG-pilG* Δ*fimL* Δ*pilH*	this study	1602
PAO1 Δ*fliC mNG-pilG* Δ*fimL* Δ*chpA*	this study	1433
PAO1 Δ*fliC mNG-pilG* Δ*fimL* Δ*chpA* Δ*pilH*	this study	1603
PAO1 Δ*fliC mNG-pilG* Δ*fimL chpA*$_{LOF}$	this study	1693
PAO1 Δ*fliC mNG-pilG* Δ*fimL chpA*$_{LOF}$ Δ*pilH*	this study	1737

PAO1 Δ*fliC mNG-pilG* Δ*pilH*	this study	969
PAO1 Δ*fliC mNG-pilG* $pilH_{LOF}$	this study	1023
PAO1 Δ*fliC mNG-pilG* $pilH_{GOF}$	this study	1404
PAO1 Δ*fliC mNG-pilG* $pilH_{GOF}$ Δ*pilA*	this study	1694
PAO1 Δ*fliC mNG-pilG* Δ*pilA*	this study	1060
PAO1 Δ*fliC mNG-pilG* Δ*pilA*	this study	1604
PAO1 Δ*fliC mNG-pilG* Δ*pilA* Δ*pilH*	this study	1727
PAO1 mNG-$pilG_{D58A}$	this study	926
PAO1 mNG-$pilG_{D58A}$ Δ*pilH*	this study	1728
PAO1 mNG-$pilG_{D58A}$ Δ*cpdA*	this study	1406
PAO1 mNG-$pilG_{D58A}$ Δ*chpA*	this study	1689
PAO1 mNG-$pilG_{D58A}$ Δ*fimL*	this study	1692
PAO1 mNG-$pilG_{D58A}$ Δ*chpA* Δ*fimL*	this study	1734
PAO1 mNG-$pilG_{D58E}$	this study	925
PAO1 mNG-$pilG_{D58E}$ Δ*pilH*	this study	1729
PAO1 mNG-$pilG_{D58E}$ Δ*cpdA*	this study	1402
PAO1 mNG-$pilG_{D58E}$ Δ*chpA*	this study	1688
PAO1 mNG-$pilG_{D58E}$ Δ*fimL*	this study	1691
PAO1 mNG-$pilG_{D58E}$ Δ*chpA* Δ*fimL*	this study	1733
PAO1 Δ*fliC mNG-pilH* Δ*chpA*	this study	1058
PAO1 Δ*fliC mNG-pilH* Δ*chpA* Δ*cpdA*	this study	1406
PAO1 Δ*fliC mNG-pilH* $chpA_{LOF}$	this study	1582
PAO1 Δ*fliC mNG-pilH* $chpA_{LOF}$ Δ*cpdA*	this study	1539
PAO1 Δ*fliC mNG-pilH* Δ*pilG*	this study	916
PAO1 Δ*fliC mNG-pilH* Δ*pilG* Δ*cpdA*	this study	1041
PAO1 Δ*fliC mNG-pilH* Δ*pilA*	this study	1605
PAO1 Δ*fliC mNG-pilH* Δ*pilA*	this study	1669
PAO1 mNG-$pilH_{LOF}$	this study	990
PAO1 mNG-$pilH_{LOF}$ Δ*cyaB*	this study	1360

PAO1 mNG-*pilH*$_{GOF}$	this study	989
PAO1 Δ*fliC* mNG-*pilH*$_{GOF}$	this study	1355
PAO1 Δ*fliC* mNG-*pilH*$_{GOF}$ Δ*pilA*	this study	1608
PAO1 Δ*fliC* mNG-*pilH*$_{GOF}$ Δ*pilA*	this study	1687
PAO1 Δ*fliC* mNG-*pilH*$_{GOF}$ Δ*cpdA*	this study	1467
PAO1 Δ*fliC* mNG-*pilH*$_{GOF}$ Δ*cpdA* Δ*chpA*	this study	1485
PAO1 Δ*fliC* mNG-*pilH*$_{GOF}$ *chpA*$_{\Delta HK}$	this study	1515
PAO1 Δ*fliC* mNG-*pilH*$_{GOF}$ *chpA*$_{\Delta HK}$ Δ*cpdA*	this study	1516
PAO1 Δ*fliC* mNG-*pilH*$_{GOF}$ *chpA*$_{LOF}$	this study	1540
PAO1 Δ*fliC* mNG-*pilH*$_{GOF}$ *chpA*$_{LOF}$ Δ*cpdA*	this study	1541
PAO1 Δ*fliC* PaQa	(Kühn *et al*, 2021)	764
PAO1 Δ*fliC* Δ*cpdA* PaQa	(Kühn *et al*, 2021)	867
PAO1 Δ*fliC* Δ*cyaB* PaQa	(Kühn *et al*, 2021)	866
PAO1 Δ*fliC* Δ*chpA* PaQa	this study	1590
PAO1 Δ*fliC* Δ*chpA* Δ*cpdA* PaQa	this study	1591
PAO1 Δ*fliC* *chpA*$_{LOF}$ PaQa	this study	1580
PAO1 Δ*fliC* *chpA*$_{LOF}$ Δ*cpdA* PaQa	this study	1581
PAO1 Δ*fliC* *pilA*$_{A86C}$ (cysteine substitution for maleimide labelling, single point mutation of chromosomal PilA)	this study	1210
PAO1 3x*Flag-pilG*	this study	HM540
PAO1 3x*Flag-pilG*$_{D58A}$ (non-phosphorylatable mutant)	this study	HM543
PAO1 3x*Flag-pilG*$_{D58E}$ (non-phosphorylatable mutant)	this study	HM545
PAO1 3x*Flag-pilG* Δ*cpdA*	this study	YI974
PAO1 3x*Flag-pilG* Δ*cyaB*	this study	HM620
PAO1 3x*Flag-pilG* Δ*pilH*	this study	HM587
PAO1 3x*Flag-pilG* *pilH*$_{LOF}$ (non-phosphorylatable mutant)	this study	HM590
PAO1 3x*Flag-pilG* *pilH*$_{GOF}$ (non-phosphorylatable mutant)	this study	HM592
PAO1 3x*Flag-pilG* *pilH*$_{GOF}$ Δ*cpdA*	this study	HM664
PAO1 PlacP1-YFP POXB20-mKate2	this study	HM413

PAO1 3x*Flag-pilG* PlacP1-YFP POXB20-mKate2	this study	HM549
PAO1 3x*Flag-pilH* PlacP1-YFP POXB20-mKate2	this study	HM438
PAO1 3xFlag-*pilH*$_{LOF}$ PlacP1-YFP POXB20-mKate2	this study	HM439
PAO1 3xFlag-*pilH*$_{GOF}$ PlacP1-YFP POXB20-mKate2	this study	HM440
PAO1 Δ*pilG* PlacP1-YFP POXB20-mKate2	this study	HM421
PAO1 Δ*pilH* PlacP1-YFP POXB20-mKate2	this study	HM422
PAO1 Δ*cpdA* PlacP1-YFP POXB20-mKate2	this study	HM419
PAO1 Δ*cyaB* PlacP1-YFP POXB20-mKate2	this study	HM416
PAO1 3x*Flag-pilG* Δ*pilH* PlacP1-YFP POXB20-mKate2	this study	HM603
PAO1 3x*Flag-pilG* Δ*cpdA* PlacP1-YFP POXB20-mKate2	this study	HM605
PAO1 3x*Flag-pilG* Δ*cyaB* PlacP1-YFP POXB20-mKate2	this study	HM628
PAO1 3x*Flag-pilG pilH*$_{LOF}$ PlacP1-YFP POXB20-mKate2	this study	HM609
PAO1 3x*Flag-pilG pilH*$_{GOF}$ PlacP1-YFP POXB20-mKate2	this study	HM611
PAO1 3x*Flag-pilG pilH*$_{GOF}$ Δ*cpdA* PlacP1-YFP POXB20-mKate2	this study	HM678
PAO1 3x*Flag-pilG*$_{D58A}$ PlacP1-YFP POXB20-mKate2	this study	HM551
PAO1 3x*Flag-pilG*$_{D58E}$ PlacP1-YFP POXB20-mKate2	this study	HM553

Appendix Table S 2: Plasmids used in this study.

**Name and relevant information**	**Source / Reference**	**Identifier**
pEX100TAP (Suicide vector based on pUC19, Amp^{R}, ColE1 ori (*E. coli*), *oriT*, *sacB*, *lacZα*)	(Schweizer & Hoang, 1995)	Na
pEX18AP (Suicide vector based on pUC18, Amp^{R}, ColE1 ori (*E. coli*), *oriT*, *sacB*, *lacZα*)	(Hoang *et al*, 1998)	Na
pEX18GM (Suicide vector based on pUC18, Gm^{R}, ColE1 ori (*E. coli*), *oriT*, *sacB*, *lacZα*)	(Hoang *et al*, 1998)	Na
pUCP18-PaQa (fluorescent reporter for cAMP level: YFP controlled by *PaQa* promoter (PA1867 and PA1868) and mKate2 controlled by *rpoD* promoter (PA0576) as reference.	(Persat *et al*, 2015)	pAP02.2
pUC18_PlacP1-YFP/POXB20-mKate2 (fluorescent reporter for cAMP level: YFP controlled by the synthetic *LacP1* promoter	this study	YI996

and mKate2 controlled by POXB20 promoter (Oxford Genetics Ltd. (UK), Sigma) as reference.		
pEx100TAP-Δ*fliC* (PA1092)	(Bertrand *et al*, 2010)	pJB215
pEx100TAP-Δ*cpdA* (PA4969)	(Inclan *et al*, 2011a)	pJTW033
pEX18GM-Δ*cpdA* (PA4969)	(Kühn *et al*, 2021)	pMK019
pEx100TAP-Δ*cyaB* (PA3217)	(Inclan *et al*, 2011a)	pJTW031
pEX18GM-Δ*cyaB* (PA3217)	(Kühn *et al*, 2021)	pMK018
pEx100TAP-Δ*pilG* (PA0408)	(Bertrand *et al*, 2010)	PJB118
pEx100TAP-Δ*pilH* (PA0409)	(Bertrand *et al*, 2010)	PJB119
pEX18AP-Δ*pilGH* (PA0408 and PA0409 including intergenic region)	this study	pXP322
pEX18GM-$chpA_{\Delta HK}$ (deletion of the histidine kinase domain of PA0413, residues 1943-2176)	this study	pMK056
pEX18GM-$chpA_{LOF}$ (insertion of the histidine kinase domain of PA0413 with substituted residues D2086A, D2087A, G2088A)	this study, based on (Bertrand *et al*, 2010)	pMK057
pEX18AP-$pilH_{LOF}$ (PA0409 with substituted residue D52A)	this study	pMK012
pEX18AP-$pilH_{GOF}$ (PA0409 with substituted residue D52E)	this study	pMK013
pEx100TAP-*mNG-pilH* (PA0409 N-terminus fused with mNeonGreen, GGGGG linker)	(Kühn *et al*, 2021)	pXP125
pEX18GM-*mNG-pilG* (PA0408 N-terminus fused with mNeonGreen, GGGGG linker)	(Kühn *et al*, 2021)	YI883
pEx100TAP-*mNG-chpA* (PA0413 N-terminus fused with mNeonGreen, GGGGG linker)	this study	pXP117
pEX18GM-$pilA_{A86C}$ (cysteine-labelled PA4525)	this study	pMK025
pEX18GM-mNG-$pilH_{LOF}$ (PA0409 with substituted residue D52A N-terminus fused with mNeonGreen, GGGGG linker)	this study	YI926
pEx100TAP-mNG-$pilH_{GOF}$ (PA0409 with substituted residue D52E N-terminus fused with mNeonGreen, GGGGG linker)	this study	HM83
pEX18GM-3x*Flag-pilG* (PA0408 N-terminus fused with 3xFlag, GGGGG linker)	this study	HM524
pEX18GM-3x*Flag*-$pilG_{D58A}$ (PA0408 D58A non-phosphorylatable mutation, N-terminus fused with 3xFlag, GGGGG linker)	this study	HM526

pEX18GM-3x*Flag-pilG*$_{D58E}$ (PA0408 D58E non-phosphorylatable mutation, N-terminus fused with 3xFlag, GGGGG linker)	this study	HM536
pEx100TAP-3x*Flag-pilH* (PA0409 N-terminus fused with 3xFlag, GGGGG linker)	this study	HM118
pEx100TAP-3xFlag-*pilH*$_{LOF}$ (PA0409 with substituted residue D52A N-terminus fused with 3xFlag, GGGGG linker)	this study	HM169
pEx100TAP-3xFlag-*pilH*$_{GOF}$ (PA0409 with substituted residue D52E N-terminus fused with 3xFlag, GGGGG linker)	this study	HM171
pEX18GM-3x*Flag-pilG* Δ*pilH (*PA0408 N-terminus fused with 3xFlag, GGGGG linker, and in-frame deletion of PA0409)	this study	HM577
pEX18GM 3x*Flag-pilG pilH*$_{LOF}$ (PA0408 N-terminus fused with 3xFlag, GGGGG linker, PA0409 with substituted residue D52A)	this study	HM583
pEX18GM 3x*Flag-pilG pilH*$_{GOF}$ (PA0408 N-terminus fused with 3xFlag, GGGGG linker, PA0409 with substituted residue D52E)	this study	HM581
pEX18GM-Δ*fimL*	this study	pMK59
pEX18GM-Δ*pilH* for *mNG-pilG*$_{D58A}$	this study	pMK60
pEX18GM-Δ*pilH* for *mNG-pilG*$_{D58E}$	this study	pMK61
pEX18GM-Δ*pilH* for *mNG-pilG*	this study	pMK62

Appendix Table S 3: Oligonucleotides used in this study.

**Identifier**	**Sequence**	**Purpose**
oXP794	ATG ACC ATG ATT ACG AAT TCC AGT TCG TGC AGC GG	Generation of pXP322
oXP795	GCT GCG ACG GGC TCA CAT GTT CGC CCT ATA TCG AC	Generation of pXP322
oXP796	TAT AGG GCG AAC ATG TGA GCC CGT CGC AGC	Generation of pXP322
oXP797	GCC TGC AGG TCG ACT CTA GAA ATG AAG GGT TGC AGT GC	Generation of pXP322
oMK136	TGC ATG CCT GCA GGT CGA CTG ATC CTG CAC ACC CTC AAG G	Generation of pMK056/57
oMK137	GGT TCA CCG ACT GCA ACT GCG AAT AGC GG	Generation of pMK056

oMK138	GCA GTT GCA GTC GGT GAA CCG GGC GCT G	Generation of pMK056
oMK139	CAG CTA TGA CCA TGA TTA CGA ACC GTC CAT GCG CGG CAT C	Generation of pMK056/57
oMK142	CCG GCC GCG GCC GCG GAG AGG GTG AGG AGG ATG	Generation of pMK057
oMK143	CTC TCC GCG GCC GCG GCC GGC ATC CGC CTC GAC	Generation of pMK057
oMK140	TGC TGG AGA ACC TCG AAC TG	Check c*hpA*$_{HK}$ locus
oMK141	TGA TCA TGA TGA TCG GCA GG	Check c*hpA*$_{HK}$ locus
oMK039	CAT GCC TGC AGG TCG ACT CAC AGA GGG ATG ACC CGG	Generation of pMK052
oMK042	GCT ATG ACC ATG ATT ACG CGG TGG AAG TGG AAG TGG	Generation of pMK052
oMK062	GAG CCG GAT TGC AAC AAG TTG GGT GTA ATT GC	Generation of pMK052
oMK063	AAC TTG TTG CAA TCC GGC TCG ACG CCG	Generation of pMK052
oLT040	GTA TCG ACC GGG CAA TTG C	Check *pilA* locus
oLT043	CTC TTG GGT GGA CTT GTC	Check *pilA* locus
YIp276	GGC GCG GCA TCA TGA TGG CGA CGA AAA TGA TGT TC	Generation of *pilG*$_{D58A}$ mutation
YIp277	GAA CAT CAT TTT CGT CGC CAT CAT GAT GCC GCG CC	Generation of *pilG*$_{D58A}$ mutation
YIp284	AGG CGC GGC ATC ATG ATT TCG ACG AAA ATG ATG TT	Generation of *pilG*$_{D58E}$ mutation
YIp285	AAC ATC ATT TTC GTC GAA ATC ATG ATG CCG CGC CT	Generation of *pilG*$_{D58E}$ mutation
YIp280	CGG GCA TGA CGA TGG CCA TCA GGA CCA CG	Generation of *pilH*$_{D52A}$ mutation
YIp281	CGT GGT CCT GAT GGC CAT CGT CAT GCC CG	Generation of *pilH*$_{D52A}$ mutation
YIp282	CCG GGC ATG ACG ATT TCC ATC AGG ACC AC	Generation of *pilH*$_{D52E}$ mutation

YIp283	GTG GTC CTG ATG GAA ATC GTC ATG CCC GG	Generation of *pilH*$_{D52E}$ mutation
hm66	GTA GTC ATC GAT TTT GTC ATC GTC TTT GTA GTC GGC GGC TTT GTC ATC GTC TTT GTA GTC GTT CGC CCT ATA TCG ACT	Generation of 3x*Flag-pilG*
hm67	TAC AAA GAC GAT GAC AAA ATC GAT GAC TAC AAA GAC GAT GAC AAA GGC GGC GGC GGC GGC ATG GAA CAG CAA TCC GAC	Generation of 3x*Flag-pilG*
hm75	TAT TTC GTG ATG GGG ATC CCA TGG CTC GTA TAA GCT TCA CCA CCA AGG ACC AG	Generation of 3x*Flag-pilG* ΔpilH
hm76	CGA CGG CCA GTG CCA AGC TTT CGG GGC TGG GCG GCA GG	Generation of 3x*Flag-pilG* ΔpilH
hm52	AAG CTT GGC ACT GGC CGTC	Generation of 3x*Flag-pilG* ΔpilH
hm53	GGG ATC CCC ATC ACG AAA TAA G	Generation of 3x*Flag-pilG* ΔpilH

Appendix Table S4: Number of measured, segmented or tracked cells for microscopy and flow cytometry experiments. If not indicated otherwise, numbers correspond to 2h data.

**Figure**	**Identifier / strain**	**Replicates**	**Number cells**
1 A	923 Δ*fliC mNG-pilG*<br>315 Δ*fliC mNG-pilH*	1<br>1	42<br>42
1 C-E	923 Δ*fliC mNG-pilG*	0h 8<br>1h 8<br>2h 8	0h 880<br>1h 1984<br>2h 4073
1 F-H	315 Δ*fliC mNG-pilH*	0h 6<br>1h 5<br>2h 6	0h 654<br>1h 846<br>2h 1930
2 A-C	*1423 ΔfliC mNG-pilG ΔchpA*<br>*1542 ΔfliC mNG-pilG chpA*$_{LOF}$	3<br>3	1403<br>1009
2 D-F	*1045 ΔfliC mNG-pilG ΔfimL*<br>*1433 ΔfliC mNG-pilG ΔfimL ΔchpA*	3<br>2	1509<br>842

3	*1055 ΔfliC mNG-pilH ΔcpdA*<br>*1406 ΔfliC mNG-pilH ΔcpdA ΔchpA*<br>*1539 ΔfliC mNG-pilH ΔcpdA* $chpA_{LOF}$	3<br>3<br>3	526<br>734<br>813
4 A, B	989 $mNG\text{-}pilH_{GOF}$ / 1355 *ΔfliC* $mNG\text{-}pilH_{GOF}$	0h 4<br>1h 4<br>2h 4	0h 432<br>1h 618<br>2h 909
4 C, D	1467 *ΔfliC* $mNG\text{-}pilH_{GOF}$ *ΔcpdA*<br>1485 *ΔfliC* $mNG\text{-}pilH_{GOF}$ *ΔcpdA ΔchpA*<br>1541 *ΔfliC* $mNG\text{-}pilH_{GOF}$ *ΔcpdA* $chpA_{LOF}$	3<br>2<br>3	639<br>618<br>788
5 C-G	337 *ΔfliC ΔcpdA*<br>342 *ΔfliC ΔcpdA ΔchpA*<br>1537 *ΔfliC ΔcpdA* $chpA_{LOF}$<br>459 *ΔfliC ΔcpdA ΔpilG*<br>1278 *ΔfliC ΔcpdA ΔpilG ΔpilH*	3<br>3<br>3<br>3<br>3	50<br>50<br>50<br>50<br>50
5 H	337 *ΔfliC ΔcpdA*<br>342 *ΔfliC ΔcpdA ΔchpA*<br>1537 *ΔfliC ΔcpdA* $chpA_{LOF}$<br>459 *ΔfliC ΔcpdA ΔpilG*<br>1278 *ΔfliC ΔcpdA ΔpilG ΔpilH*	20<br>4<br>3<br>9<br>4	30246<br>6444<br>871<br>7474<br>3700
6 A, B	1095 *ΔfliC mNG-pilH ΔcyaB*<br><br><br>916 *ΔfliC mNG-pilH ΔpilG*	0h 3<br>1h 3<br>2h 3<br>0h 2<br>1h 1<br>2h 2	0h 378<br>1h 657<br>2h 1235<br>0h 182<br>1h 177<br>2h 393
6 C, D	1055 *ΔfliC mNG-pilH ΔcpdA*<br><br><br>1041 *ΔfliC mNG-pilH ΔcpdA ΔpilG*	0h 3<br>1h 2<br>2h 3<br>0h 2<br>1h 1<br>2h 2	0h 181<br>1h 234<br>2h 526<br>0h 86<br>1h 32<br>2h 222
7 A-C	1429 *ΔfliC mNG-pilG ΔcpdA*<br>969 *ΔfliC mNG-pilG ΔpilH*<br><br>1023 *ΔfliC mNG-pilG* $pilH_{LOF}$	2h 3<br>0h 3<br>2h 3<br>0h 3<br>2h 3	2h 534<br>0h 351<br>2h 722<br>0h 265<br>2h 511

7 D-F	1404 Δ*fliC mNG-pilG* $pilH_{GOF}$	0h 3<br>1h 3<br>2h 3	0h 462<br>1h 846<br>2h 1376
8 A, B	1060/1604 Δ*fliC mNG-pilG* Δ*pilA*	0h 5<br>1h 5<br>2h 5	0h 854<br>1h 977<br>2h 1684
8 C, D	1605/1669 Δ*fliC mNG-pilH* Δ*pilA*	0h 3<br>1h 3<br>2h 3	0h 908<br>1h 854<br>2h 1082
8 E, F	969 Δ*fliC mNG-pilG* Δ*pilH*<br>1727 Δ*fliC mNG-pilG* Δ*pilA* Δ*pilH*	1h 3<br>0h 2<br>1h 2<br>2h 3	1h 625<br>0h 274<br>1h 252<br>2h 542
8 H	923 Δ*fliC mNG-pilG*<br>1060/1604 Δ*fliC mNG-pilG* Δ*pilA*<br>969 Δ*fliC mNG-pilG* Δ*pilH*<br>1023 Δ*fliC mNG-pilG* $pilH_{LOF}$<br>1727 Δ*fliC mNG-pilG* Δ*pilA* Δ*pilH*	10 min 3<br>70 min 3<br>10 min 3<br>70 min 3<br>10 min 2<br>70 min 2<br>10 min 2<br>70 min 2<br>10 min 3<br>70 min 3	10 min 575<br>70 min 1765<br>10 min 480<br>70 min 552<br>10 min 501<br>70 min 1242<br>10 min 561<br>70 min 1866<br>10 min 352<br>70 min 366
S3	312 Δ*fliC mNG-chpA*<br>*1535* Δ*fliC mNG-*$chpA_{LOF}$	2<br>2	424<br>587
S4	764 Δ*fliC* PaQa<br>*866* Δ*fliC* Δ*cyaB* PaQa<br>*867* Δ*fliC* Δ*cpdA* PaQa<br>1590 Δ*fliC* Δ*chpA* PaQa	liquid 3<br>solid 3<br>liquid 2<br>solid 2<br>liquid 2<br>solid 2<br>liquid 2<br>solid 2	liquid 1356<br>solid 672<br>liquid 532<br>solid 429<br>liquid 499<br>solid 258<br>liquid 493<br>solid 425

	1591 Δ*fliC* Δ*chpA* Δ*cpdA* PaQa<br><br>1580 Δ*fliC* *chpA*$_{LOF}$ PaQa<br><br>1581 Δ*fliC* *chpA*$_{LOF}$ Δ*cpdA* PaQa	liquid 2<br>solid 2<br>liquid 2<br>solid 2<br>liquid 2<br>solid 2	liquid 696<br>solid 316<br>liquid 491<br>solid 453<br>liquid 457<br>solid 290
S5	1098 Δ*fliC* *mNG-pilG* Δ*cyaB*<br><br><br>1429 Δ*fliC* *mNG-pilG* Δ*cpdA*	0h 3<br>1h 3<br>2h 3<br>0h 3<br>1h 3<br>2h 3	0h 372<br>1h 659<br>2h 1404<br>0h 330<br>1h 450<br>2h 534
S6	1045 Δ*fliC* *mNG-pilG* Δ*fimL*<br>1693 Δ*fliC* *mNG-pilG* Δ*fimL* *chpA*$_{LOF}$	6<br>3	2247<br>1801
S7 A,C,E,G	1098 Δ*fliC* *mNG-pilG* Δ*cyaB*<br>926 Δ*fliC* *mNG-pilG*$_{D58A}$<br>925 Δ*fliC* *mNG-pilG*$_{D58E}$	3<br>4<br>4	1404<br>1526<br>1363
S7 B,D,F,G	1429 Δ*fliC* *mNG-pilG* Δ*cpdA*<br>1403 Δ*fliC* *mNG-pilG*$_{D58A}$ Δ*cpdA*<br>1402 Δ*fliC* *mNG-pilG*$_{D58E}$ Δ*cpdA*	3<br>4<br>4	534<br>1350<br>1184
S8 A,C,E,G	926 Δ*fliC* *mNG-pilG*$_{D58A}$<br>1689 Δ*fliC* *mNG-pilG*$_{D58A}$ Δ*chpA*<br>1692 Δ*fliC* *mNG-pilG*$_{D58A}$ Δ*fimL*<br>1734 Δ*fliC* *mNG-pilG*$_{D58A}$ Δ*chpA* Δ*fimL*	4<br>2<br>2<br>2	1526<br>1206<br>1193<br>923
S8 B,D,F,G	925 Δ*fliC* *mNG-pilG*$_{D58E}$<br>1688 Δ*fliC* *mNG-pilG*$_{D58E}$ Δ*chpA*<br>1691 Δ*fliC* *mNG-pilG*$_{D58E}$ Δ*fimL*<br>1733 Δ*fliC* *mNG-pilG*$_{D58E}$ Δ*chpA* Δ*fimL*	4<br>2<br>2<br>2	1363<br>1546<br>1374<br>756
S9 A	337 Δ*fliC* Δ*cpdA*<br>459 Δ*fliC* Δ*cpdA* Δ*pilG*<br>1211 Δ*fliC* Δ*cpdA* *pilG*$_{D58A}$<br>1171 Δ*fliC* Δ*cpdA* *pilG*$_{D58E}$	20<br>9<br>3<br>3	30246<br>7474<br>866<br>1597
S9 B<br>(1000 cells<br>randomly	HM413 PlacP1-YFP	liquid 4<br>solid 5	liquid 4000<br>solid 5000

selected per<br>replicate)	HM421 Δ*pilG* PlacP1-YFP<br><br>HM549 *flag-pilG* PlacP1-YFP<br><br>HM551 *flag-pilG*$_{D58A}$ PlacP1-YFP<br><br>HM553 *flag-pilG*$_{D58E}$ PlacP1-YFP	liquid 2<br>solid 2<br>liquid 4<br>solid 5<br>liquid 3<br>solid 3<br>liquid 2<br>solid 2	liquid 2000<br>solid 2000<br>liquid 4000<br>solid 5000<br>liquid 3000<br>solid 3000<br>liquid 2000<br>solid 2000
S10<br><br><br>S10	1095 Δ*fliC mNG-pilH* Δ*cyaB*<br><br><br>1055 Δ*fliC mNG-pilH* Δ*cpdA*	3<br>3<br>3<br>3<br>2<br>3	378<br>657<br>1235<br>181<br>234<br>526
S11 A<br>spontaneous<br>reversals	177 Δ*fliC*<br>232 Δ*fliC* Δ*pilH*<br>1172 Δ*fliC pilH*$_{LOF}$<br>1155 Δ*fliC pilH*$_{GOF}$	27<br>8<br>3<br>3	31821<br>8215<br>3087<br>2278
S11 A<br>collisions	177 Δ*fliC*<br>232 Δ*fliC* Δ*pilH*<br>1172 Δ*fliC pilH*$_{LOF}$<br>1155 Δ*fliC pilH*$_{GOF}$	3<br>3<br>3<br>3	239<br>188<br>157<br>216
S11 B<br>(1000 cells<br>randomly<br>selected per<br>replicate)	HM413 PlacP1-YFP<br><br>HM422 Δ*pilH* PlacP1-YFP<br><br>HM438 *flag-pilH* PlacP1-YFP<br><br>HM439 *flag-pilH*$_{LOF}$ PlacP1-YFP<br><br>HM440 *flag-pilH*$_{GOF}$ PlacP1-YFP	liquid 4<br>solid 5<br>liquid 4<br>solid 5<br>liquid 3<br>solid 3<br>liquid 3<br>solid 3<br>liquid 3<br>solid 3	liquid 4000<br>solid 5000<br>liquid 4000<br>solid 5000<br>liquid 3000<br>solid 3000<br>liquid 3000<br>solid 3000<br>liquid 3000<br>solid 3000
S12 A-D	990 Δ*fliC mNG-pilH*$_{LOF}$	0h 3<br>1h 3<br>2h 3	0h 331<br>1h 378<br>2h 633

S12 E-H	1360 Δ*fliC mNG-pilH*$_{LOF}$ Δ*cyaB*	0h 2<br>1h 2<br>2h 2	0h 321<br>1h 434<br>2h 1044
S13	*1045 ΔfliC mNG-pilG ΔfimL*<br>*1602 ΔfliC mNG-pilG ΔfimL ΔpilH*<br><br>*1423 ΔfliC mNG-pilG ΔchpA*<br>*1603 ΔfliC mNG-pilG ΔchpA ΔpilH*<br><br>*1542 ΔfliC mNG-pilG chpA*$_{LOF}$<br>*1737 ΔfliC mNG-pilG chpA*$_{LOF}$ *ΔpilH*	6<br>2<br><br>5<br>2<br><br>3<br>2	2247<br>726<br><br>2180<br>863<br><br>1009<br>959
S15	926 Δ*fliC mNG-pilG*$_{D58A}$<br>1728 Δ*fliC mNG-pilG*$_{D58A}$ Δ*pilH*<br><br>925 Δ*fliC mNG-pilG*$_{D58E}$<br>1729 Δ*fliC mNG-pilG*$_{D58E}$ Δ*pilH*	4<br>2<br><br>4<br>2	1526<br>1173<br><br>1363<br>892
S16	1608/1687 Δ*fliC mNG-pilH*$_{GOF}$ Δ*pilA*<br><br><br><br>1694 Δ*fliC mNG-pilG* Δ*pilA pilH*$_{GOF}$	0h 2<br>1h 2<br>2h 2<br><br>0h 3<br>1h 3<br>2h 3	0h 261<br>1h 237<br>2h 176<br><br>0h 561<br>1h 704<br>2h 978

References for Appendix

Barken KB, Pamp SJ, Yang L, Gjermansen M, Bertrand JJ, Klausen M, Givskov M, Whitchurch CB, Engel JN & Tolker-Nielsen T (2008) Roles of type IV pili, flagellum-mediated motility and extracellular DNA in the formation of mature multicellular structures in *Pseudomonas aeruginosa* biofilms. *Environmental Microbiology* 10: 2331–2343

Bertrand JJ, West JT & Engel JN (2010) Genetic Analysis of the Regulation of Type IV Pilus Function by the Chp Chemosensory System of Pseudomonas aeruginosa. *Journal of Bacteriology* 192: 994–1010

Fulcher NB, Holliday PM, Klem E, Cann MJ & Wolfgang MC (2010) The Pseudomonas aeruginosa Chp chemosensory system regulates intracellular cAMP levels by modulating adenylate cyclase activity: Regulation of cAMP by a chemosensory system. *Molecular Microbiology* 76: 889–904

Hoang TT, Karkhoff-Schweizer RR, Kutchma AJ & Schweizer HP (1998) A broad-host-range Flp-FRT recombination system for site-specific excision of chromosomally-located DNA sequences: application for isolation of unmarked Pseudomonas aeruginosa mutants. *Gene* 212: 77–86

Holloway BW & Morgan AF (1986) Genome Organization in Pseudomonas. *Annu Rev Microbiol* 40: 79–105

Inclan YF, Huseby MJ & Engel JN (2011a) FimL Regulates cAMP Synthesis in Pseudomonas aeruginosa. *PLoS ONE* 6: e15867

Inclan YF, Huseby MJ & Engel JN (2011b) FimL Regulates cAMP Synthesis in Pseudomonas aeruginosa. *PLoS ONE* 6: e15867

Kühn MJ, Talà L, Inclan YF, Patino R, Pierrat X, Vos I, Al-Mayyah Z, Macmillan H, Negrete J, Engel JN, *et al* (2021) Mechanotaxis directs *Pseudomonas aeruginosa* twitching motility. *Proc Natl Acad Sci USA* 118: e2101759118

Persat A, Inclan YF, Engel JN, Stone HA & Gitai Z (2015) Type IV pili mechanochemically regulate virulence factors in Pseudomonas aeruginosa. *Proceedings of the National Academy of Sciences of the United States of America* 112: 7563–7568

Schweizer HP & Hoang TT (1995) An improved system for gene replacement and xylE fusion analysis in Pseudomonas aeruginosa. *Gene* 158: 15–22

Whitchurch CB, Beatson SA, Comolli JC, Jakobsen T, Sargent JL, Bertrand JJ, West J, Klausen M, Waite LL, Kang PJ, *et al* (2005) Pseudomonas aeruginosa fimL regulates multiple virulence functions by intersecting with Vfr-modulated pathways: FimL is required for multiple virulence functions. *Molecular Microbiology* 55: 1357–1378
