# Supplementary material for: Two antagonistic response regulators control Pseudomonas aeruginosa polarization during mechanotaxis
Source: EMBO J. 2023 Feb 16;42(7):e112165. doi: 10.15252/embj.2022112165 (PMC10519157; doi:10.15252/embj.2022112165)
Supplement: Supplementary file 2 — Movie EV1 [file EMBJ-42-e112165-s001.zip › Movie EV1.docx]

**Movie EV1: Single twitching *Pseudomonas aeruginosa* cell with fluorescently labelled type IV pili.** During the reversal of twitching direction T4P disappear at the initially leading cell pole (upper pole) and reappear at the opposite cell pole which becomes the new leading cell pole. Timestamp, min:sec; scale bar, 2 µm.
