# Supplementary material for: Two antagonistic response regulators control Pseudomonas aeruginosa polarization during mechanotaxis
Source: EMBO J. 2023 Feb 16;42(7):e112165. doi: 10.15252/embj.2022112165 (PMC10519157; doi:10.15252/embj.2022112165)
Supplement: Supplementary file 3 — Movie EV2 [file EMBJ-42-e112165-s006.zip › Movie EV2.docx]

**Movie EV2: Single cell twitching of hyper-reversing *pilG* and *chpA* mutants.** Twitching cells were recorded at the interface between agarose and a glass coverslip after 2h incubation at 37°C. To bypass low cAMP level and rescue twitching, *cpdA* was deleted in all mutants. To ensure that cells are not moving by flagellar motility, *fliC* was deleted in all mutants. The reference mutant Δ*cpdA* twitches directionally and reverses frequently. In contrast, all other shown mutants hyper-reverse, i.e. cells constantly alternate between forward and reverse twitching rendering them unable to move persistently in a given direction. Each movie is sped up 125 times and repeated 3 times. Timestamp, min:sec; scale bar, 10 µm.
