# Supplementary material for: Two antagonistic response regulators control Pseudomonas aeruginosa polarization during mechanotaxis
Source: EMBO J. 2023 Feb 16;42(7):e112165. doi: 10.15252/embj.2022112165 (PMC10519157; doi:10.15252/embj.2022112165)
Supplement: Supplementary file 4 — Movie EV3 [file EMBJ-42-e112165-s005.zip › Movie EV3.docx]

**Movie EV3: Single cell twitching of *pilG* point mutants in Δ*pilH*.** Deletion of *pilH* has no effect on the hyper-reversing phenotype in mutants with non-functional and non-phosphorylatable PilG. Twitching cells were recorded at the interface between agarose and a glass coverslip after 2h incubation at 37°C. To bypass low cAMP level and rescue twitching, *cpdA* was deleted in all mutants. To ensure that cells are not moving by flagellar motility, *fliC* was deleted in all mutants. Each movie is sped up 125 times and repeated 6 times. Timestamp, min:sec; scale bar, 10 µm.
