# Supplementary material for: Two antagonistic response regulators control Pseudomonas aeruginosa polarization during mechanotaxis
Source: EMBO J. 2023 Feb 16;42(7):e112165. doi: 10.15252/embj.2022112165 (PMC10519157; doi:10.15252/embj.2022112165)
Supplement: Supplementary file 5 — Movie EV4 [file EMBJ-42-e112165-s004.zip › Movie EV4.docx]

**Movie EV4: Pole-to-pole movement of mNG-PilG signal over 3 min.** The images were recorded 10 min after surface contact allowing the agar pad to settle down to reduce the drift of cells during recording. Images also used in Figure 8G. Scale bar, 2 µm.
