# Supplementary material for: Two antagonistic response regulators control Pseudomonas aeruginosa polarization during mechanotaxis
Source: EMBO J. 2023 Feb 16;42(7):e112165. doi: 10.15252/embj.2022112165 (PMC10519157; doi:10.15252/embj.2022112165)
Supplement: Supplementary file 6 [file EMBJ-42-e112165-s003.pdf]

THE EMBO JOURNAL

Two antagonistic response regulators control Pseudomonas aeruginosa polarization during mechanotaxis

Marco Kühn, Henriette Macmillan, Lorenzo Talà, Yuki Inclan, Ramiro Patino, Xavier Pierrat, Zainebe Al-Mayyah, Joanne Engel, and Alexandre Persat
DOI: 10.15252/embj.2022112165

Corresponding author(s): Alexandre Persat (alexandre.persat@epfl.ch) , Joanne Engel (Joanne.Engel@ucsf.edu)

Review Timeline:

Submission Date:	20th Jul 22
Editorial Decision:	30th Aug 22
Revision Received:	30th Nov 22
Editorial Decision:	11th Jan 23
Revision Received:	24th Jan 23
Accepted:	30th Jan 23

Editor: Ieva Gailite

Transaction Report:

(Note: With the exception of the correction of typographical or spelling errors that could be a source of ambiguity, letters and reports are not edited. Depending on transfer agreements, referee reports obtained elsewhere may or may not be included in this compilation. Referee reports are anonymous unless the Referee chooses to sign their reports.)

1st Editorial Decision **30th Aug 2022**

Thank you for submitting your manuscript for consideration by the EMBO Journal. I sincerely apologise for the protracted assessment of your manuscript due to delayed submission of referee reports. We have now received comments from three reviewers, which are included below for your information.

As you will see from the reports, the reviewers appreciate the work, while also indicating a number of aspects where they would require further experimental support, in particular on PilG phosphorylation and its impact on PilG localisation (referees #1 and #3). Reviewer #2 also raises the question of potential differential PilG regulation in liquid cultures vs upon surface attachment. Furthermore, reviewer #2 requests some insight into the direct interactions between PilG, PilH and ChpA. I find the reviewer comments generally reasonable. Therefore, based on these broadly positive assessments, I would like to invite you to address the issues raised by the reviewers in a revised manuscript. I would be happy to discuss the revision and the feasibility of individual experiments in more detail via email or phone/videoconferencing - please let me know which option you prefer.

We generally allow three months as standard revision time. As a matter of policy, competing manuscripts published during this period will not negatively impact on our assessment of the conceptual advance presented by your study. However, please contact me as soon as possible upon publication of any related work to discuss the appropriate course of action. Should you foresee a problem in meeting this three-month deadline, please contact us to arrange an extension.

When preparing your letter of response to the referees' comments, please bear in mind that this will form part of the Review Process File and will therefore be available online to the community. For more details on our Transparent Editorial Process, please visit our website: https://www.embopress.org/page/journal/14602075/authorguide#transparentprocess. Please also see the attached instructions for further guidelines on preparation of the revised manuscript.

Please feel free to contact me if you have any further questions regarding the revision. Thank you for the opportunity to consider your work for publication, and I look forward to your revision.

Referee #1:

This study advances an understanding of the molecular mechanism of Chp-dependent regulation of T4P in Pseudomonas aeruginosa. Prior work had established that the PilG and PilH were antagonistic response regulators that influence Chp-dependent mechanotaxis, and the current study aimed to identify the molecular mechanism by which these proteins antagonized one another. Previous work suggested that PilH was necessary for induced reversals during twitching motility, but the current study very nicely demonstrates that PilH is not inherently critical for inducing reversals - it largely promotes reversals by antagonizing PilG. The authors also demonstrate that phosphorylated PilH is necessary to promote this antagonism, and that PilH-P correlates with a reduction in the PilG phosphorylation. Thus, suggesting that PilH antagonizes PilG by preventing its phosphorylation by ChpA. The authors also establish a role for ChpA in the localization of PilG and PilH upon surface contact. The work presented is well done and clearly presented. But the model presented is not thoroughly tested - especially with regards to testing the effect of PilG phosphorylation on the phenotypes studied. Specific comments are listed below:

Why is an asymmetry index not shown for ΔfimL ΔchpA in Fig. 2F? This index should be calculated to demonstrate that there is no asymmetric localization of PilG in the absence of polar localization.

Line 238 - delete "upon"

The model proposed suggests that phosphorylation of PilG is the main output of the Chp system. This should be formally tested by making a phosphomimetic mutant D to E mutant of PilG (PilG-GOF). This PilG-GOF allele should presumably rescue mechanotaxis in the ChpA-LOF mutant to resemble the phenotype of the pilH deletion of pilH-LOF mutant. This could also be tested in the cpdA mutant background to ensure that reduced cAMP levels are not confounding the analysis.

Also, the model would presume that a constitutively phosphorylated PilG would be insensitive to regulation by PilH. This should be tested by placing the PilG-GOF allele in a background with ChpA-LOF and PilH-GOF. Despite the presence of PilH-GOF, this strain should phenocopy a pilH deletion mutant if the main function of activated PilH is to prevent PilG phosphorylation.

In a similar vein, it would be valuable to demonstrate that PilH cannot alter Chp dependent effects (mechanotaxis and PilG polar localization / asymmetry) when PilG cannot be phosphorylated by creating and testing a PilG D to A mutant (PilG-LOF).

Referee #2:

The report by Kuhn et al. revealed how P. aeruginosa control twitching direction in response to mechanosensing signals using two antagonistic response regulators, PilG and PilH. The authors found that while PilG is required for the reversal of twitching direction, PilH antagonizes PilG by controlling its phosphorylation. The experiments are overall well designed and executed. Especially, the authors used nice control experiments to exclude the interference from cAMP.

My major questions about this report:

1. The evidence from fluorescence microscopy and biochemical experiments strongly suggests that ChpA, PilG, and PilH interact with each other and that PilH could even compete with PilG on binding to ChpA. However, no evidence, even preliminary evidence was shown in the manuscript. The reviewer believes that some evidence on protein interaction is required to support the central conclusions of this report.
2. In the presence of PilHGOF, PilG initially localizes more asymmetrically then loses polarity on surface (Fig. 6), which suggests that PilH regulates PilG differently in liquid culture and on solid surface. How does mechanosensing regulate PilG in this case, and will PilHGOF still regulate PilG in a mutant with pilus defect? In addition, this result is contradictory to the authors' hypothesis in Line 120 - 121.

I have a few minor suggestions on the writing and data presentation:

1. The authors claimed that PilH is polar localized but not polarized. However, I don't see this from Fig. 1B. if you look at the fluorescence intensities at cell poles (only at poles) in the 2 h data, you won't find significant difference between PilG and PilH.
2. The trajectories in Fig. 4C are very difficult to read, even with the help of three highlighted track. It will be more convincing if the authors present reversal frequencies directly.
3. Line 106 - 117. The definitions of polar index and asymmetry index should be clarified here.
4. Line 207. No evidence for this, yet.
5. Line 238. Grammar problem.
6. Line 448 - 449. Please provide the information on how the "circular polar area" is defined.
7. Line 451. "index of 0, the fluorescence"...
8. Line 463 - 465. Please provide more information on the correction method, as well as the evidence on "more accurate".
9. Throughout the text, figures and figure legend, when presented as genotype, PilHGOF should be pilHGOF (italic). The same thing applies to PilG, ChpA, etc.
10. Fig. 2E, 2F. Why panel F only contains the data from two strains, while 2E contains 3?

Referee #3:

The manuscript by Kuhn and colleagues addresses the question of how PilG and PilH function in Chp/T4P mechanosensing in Pseudomonas aeruginosa. The experiments combine genetic mutants and quantitative microscopy to address the roles of protein localization and phosphorylation in controlling T4P assembly at one or another bacterial pole. The results are specific to the P. aeruginosa system, but suggest how two response regulators can function in a chemotaxis system.

In general, the conclusions are supported by the data that are presented, although the role of PilG phosphorylation in localization/polar retention would be better tested directly (through the use of phosphoablative and phosphomimetic point mutants), as the experiments carried out with a ChpA LOF demonstrate that ChpA (as well as FimL) play roles in localization of PilG that are likely independent of its phosphorylation state.

The authors make a point of stating that PilG-P must be restricted to the leading pole (Line 295-6) to polarize motility. It seems equally probable that the phosphorylation of PilG could be limited to the leading pole (because this is where the activating signal is present via ChpA/PilJ), and that ensuring a short half-life for PilG-P (by having PilH-P also present) would "reset" the system so that T4P retraction would again create a "pro-assembly" signal and generate a feed forward loop.

Lastly, the authors clearly indicate the number of biological replicates used to generate most of the figures that are presented in the paper, but the actual number of measurements per biological replicate & experiment is never indicated. This information should be provided as part of the data.

Minor points-
Line 226 - "PilG-mNG"?
Line 243 - "recruitment in all three mutants" - 2 mutants plus WT?

Two antagonistic response regulators control *Pseudomonas aeruginosa* polarization during mechanotaxis.

Marco J. Kühn[1], Henriette Macmillan[2], Lorenzo Talà[1], Yuki Inclan[2], Ramiro Patino[2], Xavier Pierrat[1], Zainebe Al-Mayyah[1], Joanne N. Engel[*,2,3] and Alexandre Persat[*,1]

[1]Institute of Bioengineering and Global Health Institute, School of Life Sciences, EPFL, Lausanne, Switzerland

[2]Department of Medicine, University of California, San Francisco, USA

[3]Department of Microbiology and Immunology, University of California, San Francisco, USA

*Correspondence to: alexandre.persat@epfl.ch, joanne.engel@ucsf.edu

Response to reviewers

Referee #1:

This study advances an understanding of the molecular mechanism of Chp-dependent regulation of T4P in Pseudomonas aeruginosa. Prior work had established that the PilG and PilH were antagonistic response regulators that influence Chp-dependent mechanotaxis, and the current study aimed to identify the molecular mechanism by which these proteins antagonized one another. Previous work suggested that PilH was necessary for induced reversals during twitching motility, but the current study very nicely demonstrates that PilH is not inherently critical for inducing reversals - it largely promotes reversals by antagonizing PilG. The authors also demonstrate that phosphorylated PilH is necessary to promote this antagonism, and that PilH-P correlates with a reduction in the PilG phosphorylation. Thus, suggesting that PilH antagonizes PilG by preventing its phosphorylation by ChpA. The authors also establish a role for ChpA in the localization of PilG and PilH upon surface contact. The work presented is well done and clearly presented. But the model presented is not thoroughly tested - especially with regards to testing the effect of PilG phosphorylation on the phenotypes studied. Specific comments are listed below:

- We thank the reviewer for her/his/their positive comments.

Why is an asymmetry index not shown for ΔfimL ΔchpA in Fig. 2F? This index should be calculated to demonstrate that there is no asymmetric localization of PilG in the absence of polar localization.

- We thank the reviewer for raising this point. We included the missing analysis in Figure 2F and modified the caption accordingly, where we note that the asymmetry index of proteins that do not localize to the poles should be interpreted with caution.

Line 238 - delete "upon"

- Deleted.

The model proposed suggests that phosphorylation of PilG is the main output of the Chp system. This should be formally tested by making a phosphomimetic mutant D to E mutant of PilG (PilG-GOF). This PilG-GOF allele should presumably rescue mechanotaxis in the ChpA-LOF mutant to resemble the phenotype of the pilH deletion of pilH-LOF mutant. This

could also be tested in the cpdA mutant background to ensure that reduced cAMP levels are not confounding the analysis.

- We thank the reviewer for raising this important point. We therefore performed extensive characterization of the PilG phosphomimetic mutants. Our experiments however showed an important limitation: the expected gain-of-function mutation of PilG (catalytic aspartate to glutamate) yields a loss-of-function phenotype with respect to twitching motility. Failing to generate gain-of-function mutants by D to E mutation of the catalytic site is well-documented (see references Smith *et al*, 2004; Guzzo *et al*, 2018).
- Despite the limitations in functionality of $pilG_{GOF}$, we now report protein localization, twitching behaviour and cAMP levels for $pilG_{D58A}$ and $pilG_{D58E}$ (lines 178-1921, Appendix Figures S7-S9). These experiments raised important points regarding the protein localization patterns of $PilG_{D58A}$ and $PilG_{D58E}$. In particular, we found both PilG mutants still get recruited by both FimL and ChpA. We conclude that the mutations are not entirely limiting their ability to interact with other Chp components. We hypothesize that the GOF mutation impacts PilG's downstream function with the extension machinery. To test this possibility, we have to identify PilG's target first, which is beyond the scope of this manuscript.

- Due to the addition of PilG point mutant data, we reshuffled the order of the results for clarity; we moved the PilH point mutant data further up (to lines 217-236). The effect of $PilH_{GOF}$ on PilG remains at its previous location near the end of the results section.

Also, the model would presume that a constitutively phosphorylated PilG would be insensitive to regulation by PilH. This should be tested by placing the PilG-GOF allele in a background with ChpA-LOF and PilH-GOF. Despite the presence of PilH-GOF, this strain should phenocopy a pilH deletion mutant if the main function of activated PilH is to prevent PilG phosphorylation.

- We agree that this would be an elegant experiment to demonstrate the function of PilH. Due to limitation in PilG point mutations, we instead validated that PilH-dependent regulation of PilG polar localization requires specifically functional ChpA but not FimL (lines 291-293, Appendix Figure S13). Together with the PhosTag data from Figure 7, this demonstrates that PilH functions through regulation of ChpA-dependent phosphorylation of PilG.
- Nevertheless, we tested whether deletion of *pilH* has any effect on polar localization of non-phosphorylatable $PilG_{D58A}$ and $PilG_{D58E}$ or the twitching behaviour of $pilG_{D58A}$ and $pilG_{D58E}$ mutants (lines 313-318, Appendix Figure S15, Movie EV3). We found that both non-phosphorylatable PilG mutants are insensitive to regulation by PilH.

In a similar vein, it would be valuable to demonstrate that PilH cannot alter Chp dependent effects (mechanotaxis and PilG polar localization / asymmetry) when PilG cannot be phosphorylated by creating and testing a PilG D to A mutant (PilG-LOF).

- We thank the reviewer for this comment. We performed the suggested experiments along with testing $PilG_{D58E}$ (see answers to the two previous points).

Referee #2:

The report by Kuhn et al. revealed how P. aeruginosa control twitching direction in response to mechanosensing signals using two antagonistic response regulators, PilG and PilH. The authors found that while PilG is required for the reversal of twitching direction, PilH antagonizes PilG by controlling its phosphorylation. The experiments are overall well designed and executed. Especially, the authors used nice control experiments to exclude the interference from cAMP.

- We thank the reviewer for the positive comments.

My major questions about this report:

1. The evidence from fluorescence microscopy and biochemical experiments strongly suggests that ChpA, PilG, and PilH interact with each other and that PilH could even compete with PilG on binding to ChpA. However, no evidence, even preliminary evidence was shown in the manuscript. The reviewer believes that some evidence on protein interaction is required to support the central conclusions of this report.

- We thank the reviewer for raising this important point, which can be taken for granted given the demonstrated interactions of histidine kinase and response regulators in Che chemotaxis system. A previous study demonstrated that purified ChpA can separately phosphorylate PilG and PilH *in vitro* (Silversmith *et al*, 2016). We believe this is strong evidence for a direct interaction of ChpA with PilG, and ChpA with PilH. We previously published results from AP-MS of PilG which allowed us to identify FimL as a stable interactor (Inclan *et al*, 2016). Identifying the entire interaction network of the Chp system is for us a long-term goal, and will be the topic of future studies.
- Because ChpA-PilG-PilH interactions are so central to our conclusions, we clarified the manuscript with the following (lines 232-236):
  "Although direct interaction between PilH forms and ChpA has yet to be demonstrated, interactions between response regulators and histidine kinases are well established in homologous chemosensory systems (Kentner & Sourjik, 2009). Regarding Chp, *in vitro* phosphorylation of purified PilG and PilH by ChpA strongly suggests direct interaction of ChpA with both PilG and PilH (Silversmith *et al*, 2016)."

2. In the presence of PilHGOF, PilG initially localizes more asymmetrically then loses polarity on surface (Fig. 6), which suggests that PilH regulates PilG differently in liquid culture and on solid surface. How does mechanosensing regulate PilG in this case, and will PilHGOF still regulate PilG in a mutant with pilus defect? In addition, this result is contradictory to the authors' hypothesis in Line 120 - 121.

- We thank the reviewer for pointing out this aspect which we initially neglected. This comment motivated us to investigate PilG activation in liquid vs solid surfaces. We compared different PilG localisation patterns in liquid, surface associated cells and in a *pilA* deletion background, which in our model should reproduce a liquid state without mechanical input from T4P. Those results are part of a new paragraph and a new main figure at the end of the results section (lines 319-347, Figure 8, Appendix Figure S16).
- In a nutshell, our results show that PilG is always polarized irrespectively of being in liquid or on surface. Also, PilH regulates PilG polarization under all conditions. Figure 1G indicates that there is even some kind of homeostasis of PilG polarization, despite increasing surface sensing (Persat *et al*, 2015) and increasing PilH

recruitment (Figure 1). **The main change induced by mechanosensing is in the persistence of polarization in one direction**. PilG polarity frequently switches in cells from liquid culture or in *pilA* deletion mutants. PilG polarity only stabilizes over time, which is dependent on mechanical input, thereby requiring PilA. In Δ*pilH,* PilG is locked at one pole, independently of PilA. We conclude that Chp is always on in liquid or surface, but that mechanosensing biases which pole has active Chp to sustain local positive feedback. This spatial control of activation maintains PilG polarization. According to the new results, we modified the discussion in lines 357-359, 364-371 and 428-432.

- Line 120-121: Stating this hypothesis that we then proved wrong was just stylistic. To clarify that this is not our conclusion, we modified the text in lines 132-134: "Sustained PilG and PilH phosphorylation by ChpA during surface growth could potentially explain these changes in localization. We thus went on to identify the function of ChpA in polar localization of the two response regulators."

I have a few minor suggestions on the writing and data presentation:

1. The authors claimed that PilH is polar localized but not polarized. However, I don't see this from Fig. 1B. if you look at the fluorescence intensities at cell poles (only at poles) in the 2 h data, you won't find significant difference between PilG and PilH.

- This first panel of Figure 1 was intended to give a visual representation of the vastly different localization pattern of PilG and PilH. It was however performed on only a selected subset of cells which gave rise to artefact from analysis. To prevent confusion, we only show one timepoint of the average maps as a visual guide, and clearly refer to the proper population-wide quantification in the text when making claims about quantitative localization (lines 114-120).

2. The trajectories in Fig. 4C are very difficult to read, even with the help of three highlighted track. It will be more convincing if the authors present reversal frequencies directly.

- We understand the reviewer's point. In addition to the trajectories, we now provide representative movies (Movie EV2) along with quantification of reversal rates (Figure 5H). For clarity, we moved the *pilH* reversals to Appendix Figure S11, since this was not relevant in this context and is redundant with a previous publication (Kühn *et al*, 2021).

3. Line 106 - 117. The definitions of polar index and asymmetry index should be clarified here.

- We extended our explanation now in lines 119-123.
- We also included very clear definitions in the Methods section "Polar Localization Index" and "Asymmetry Index" (starting line 552 and 579, respectively).

4. Line 207. No evidence for this, yet.

- True, we modified the text (lines 259-261).

5. Line 238. Grammar problem.

- Fixed.

6. Line 448 - 449. Please provide the information on how the "circular polar area" is defined.

- We extended the respective Methods section and clarified the definition (lines 554-558).

7. Line 451. "index of 0, the fluorescence"...

- Fixed.

8. Line 463 - 465. Please provide more information on the correction method, as well as the evidence on "more accurate".

- We provide more information about the correction method and explain the procedure how we estimate the accuracy of our quantification and different correction methods used previously and in the current manuscript (lines 556-576).

9. Throughout the text, figures and figure legend, when presented as genotype, PilHGOF should be pilHGOF (italic). The same thing applies to PilG, ChpA, etc.

- We often refer to the respective proteins, however, when clearly referring to the genotype we display gene names now as mentioned by the reviewer.

10. Fig. 2E, 2F. Why panel F only contains the data from two strains, while 2E contains 3?

- See answer to reviewer 1. We added the missing data.

Referee #3:

The manuscript by Kuhn and colleagues addresses the question of how PilG and PilH function in Chp/T4P mechanosensing in Pseudomonas aeruginosa. The experiments combine genetic mutants and quantitative microscopy to address the roles of protein localization and phosphorylation in controlling T4P assembly at one or another bacterial pole. The results are specific to the P. aeruginosa system, but suggest how two response regulators can function in a chemotaxis system.

In general, the conclusions are supported by the data that are presented, although the role of PilG phosphorylation in localization/polar retention would be better tested directly (through the use of phosphoablative and phosphomimetic point mutants), as the experiments carried out with a ChpA LOF demonstrate that ChpA (as well as FimL) play roles in localization of PilG that are likely independent of its phosphorylation state.

- We thank the reviewer for his/her/their comment. We refer to the response to reviewer 1 regarding the phosphomimetic mutations.

The authors make a point of stating that PilG-P must be restricted to the leading pole (Line 295-6) to polarize motility. It seems equally probable that the phosphorylation of PilG could be limited to the leading pole (because this is where the activating signal is present via ChpA/PilJ), and that ensuring a short half-life for PilG-P (by having PilH-P also present)

would "reset" the system so that T4P retraction would again create a "pro-assembly" signal and generate a feed forward loop.

- We also think this is a valid hypothesis and added a line do the discussion (lines 381-383).

Lastly, the authors clearly indicate the number of biological replicates used to generate most of the figures that are presented in the paper, but the actual number of measurements per biological replicate & experiment is never indicated. This information should be provided as part of the data.

- We added Appendix Table S4 containing the number of measured and tracked cells.

Minor points-

Line 226 - "PilG-mNG"?

- It should say mNG-PilG.

Line 243 - "recruitment in all three mutants" - 2 mutants plus WT?

- Modified as suggested.

1st Revision - Editorial Decision 11th Jan 2023

Thank you for submitting a revised version of your manuscript. I sincerely apologise for the delay in communicating the decision due to the holiday period. Your study has now been seen by all original referees, who find that their previous concerns have been addressed and now recommend publication of the manuscript. There remain only a couple of minor editorial points that have to be addressed before I can extend formal acceptance of the manuscript.

Please let me know if you have any further questions regarding any of these points. You can use the link below to upload the revised files.

Thank you again for giving us the chance to consider your manuscript for The EMBO Journal. I look forward to receiving the final version.

Referee #1:

The authors have expertly responded to all of my comments raised during the first round of review. It is unfortunate that the PilG D58E mutant is nonfunctional. However, there are no other facile alternatives to generate this type of allele that I am aware. All of the data presented are certainly consistent with the model proposed. So I have no other comments and would like to simply congratulate the authors on the very nice study that they have put together.

Referee #2:

The authors have addressed all my concerns.

Referee #3:

The revised manuscript by Kuhn et al. provides additional experimental data that by and large addresses the questions and comments of the initial review. The authors provide a model for how a positive feed-forward loop can maintain persistent directional twitching, and demonstrate a role for PilH in breaking that feedback loop via a mechanism that is sensitive to PilH-phosphorylation (conformation) but not PilH phosphotransfer per se. The findings provide a solid foundation upon which to build understanding of how these proteins might function in the setting of taxis toward chemical stimuli, also described in the literature but not considered in this current work.

Minor comment - line 304, the slower migrating band corresponds to the phosphorylated form of PilG.

All editorial and formatting issues were resolved by the authors.

Thank you for addressing the final editorial issues. I am now pleased to inform you that your manuscript has been accepted for publication.

EMBO Press Author Checklist

Corresponding Author Name: Alexandre Persat, Joanne Engel	
Journal Submitted to: The EMBO Journal	
Manuscript Number: EMBOJ-2022-112165	

USEFUL LINKS FOR COMPLETING THIS FORM
The EMBO Journal - Author Guidelines
EMBO Reports - Author Guidelines
Molecular Systems Biology - Author Guidelines
EMBO Molecular Medicine - Author Guidelines

Reporting Checklist for Life Science Articles (updated January
This checklist is adapted from Materials Design Analysis Reporting (MDAR) Checklist for Authors. MDAR establishes a minimum set of requirements in transparent reporting in the life sciences (see Statement of Task: 10.31222/osf.io/9sm4x). Please follow the journal's guidelines in preparing your
Please note that a copy of this checklist will be published alongside your article.

Abridged guidelines for figures
1. Data
The data shown in figures should satisfy the following conditions:
- the data were obtained and processed according to the field's best practice and are presented to reflect the results of the experiments in an accurate and unbiased manner.
- ideally, figure panels should include only measurements that are directly comparable to each other and obtained with the same assay.
- plots include clearly labeled error bars for independent experiments and sample sizes. Unless justified, error bars should not be shown for technical
- if n<5, the individual data points from each experiment should be plotted. Any statistical test employed should be justified.
- Source Data should be included to report the data underlying figures according to the guidelines set out in the authorship guidelines on Data

2. Captions
Each figure caption should contain the following information, for each panel where they are relevant:
- a specification of the experimental system investigated (eg cell line, species name).
- the assay(s) and method(s) used to carry out the reported observations and measurements.
- an explicit mention of the biological and chemical entity(ies) that are being measured.
- an explicit mention of the biological and chemical entity(ies) that are altered/varied/perturbed in a controlled manner.
- the exact sample size (n) for each experimental group/condition, given as a number, not a range;
- a description of the sample collection allowing the reader to understand whether the samples represent technical or biological replicates (including how many animals, litters, cultures, etc.).
- a statement of how many times the experiment shown was independently replicated in the laboratory.
- definitions of statistical methods and measures:
  - common tests, such as t-test (please specify whether paired vs. unpaired), simple χ2 tests, Wilcoxon and Mann-Whitney tests, can be unambiguously identified by name only, but more complex techniques should be described in the methods section;
  - are tests one-sided or two-sided?
  - are there adjustments for multiple comparisons?
  - exact statistical test results, e.g., P values = x but not P values < x;
  - definition of 'center values' as median or average;
  - definition of error bars as s.d. or s.e.m.

Please complete ALL of the questions below.
Select "Not Applicable" only when the requested information is not relevant for your study.

Materials

Newly Created Materials	Information included in the manuscript?	In which section is the information available? (Reagents and Tools Table, Materials and Methods, Figures, Data Availability Section)
New materials and reagents need to be available; do any restrictions apply?	Yes	Materials and Methods, Appendix Tables S1-S3

Antibodies	Information included in the manuscript?	In which section is the information available? (Reagents and Tools Table, Materials and Methods, Figures, Data Availability Section)
For **antibodies** provide the following information:<br>- Commercial antibodies: RRID (if possible) or supplier name, catalogue number and or/clone number<br>- Non-commercial: RRID or citation	Yes	Materials and Methods

DNA and RNA sequences	Information included in the manuscript?	In which section is the information available? (Reagents and Tools Table, Materials and Methods, Figures, Data Availability Section)
**Short novel DNA or RNA including primers, probes**: provide the sequences.	Yes	Materials and Methods, Appendix Table S3

Cell materials	Information included in the manuscript?	In which section is the information available? (Reagents and Tools Table, Materials and Methods, Figures, Data Availability Section)
**Cell lines:** Provide species information, strain. Provide accession number in repository **OR** supplier name, catalog number, clone number, and/**OR** RRID.	Not Applicable	
**Primary cultures:** Provide species, strain, sex of origin, genetic modification status.	Not Applicable	
Report if the cell lines were recently **authenticated** (e.g., by STR profiling) and tested for mycoplasma contamination.	Not Applicable	

Experimental animals	Information included in the manuscript?	In which section is the information available? (Reagents and Tools Table, Materials and Methods, Figures, Data Availability Section)
**Laboratory animals or Model organisms:** Provide species, strain, sex, age, genetic modification status. Provide accession number in repository **OR** supplier name, catalog number, clone number, **OR** RRID.	Not Applicable	
**Animal observed in or captured from the field:** Provide species, sex, and age where possible.	Not Applicable	
Please detail **housing and husbandry conditions**.	Not Applicable	

Plants and microbes	Information included in the manuscript?	In which section is the information available? (Reagents and Tools Table, Materials and Methods, Figures, Data Availability Section)
**Plants:** provide species and strain, ecotype and cultivar where relevant, unique accession number if available, and source (including location for collected wild specimens).	Not Applicable	
**Microbes:** provide species and strain, unique accession number if available, and source.	Yes	Materials and Methods, Appendix Table S1

Human research participants	Information included in the manuscript?	In which section is the information available? (Reagents and Tools Table, Materials and Methods, Figures, Data Availability Section)
If collected and within the bounds of privacy constraints report on age, sex and gender or ethnicity for all study participants.	Not Applicable	

Core facilities	Information included in the manuscript?	In which section is the information available? (Reagents and Tools Table, Materials and Methods, Figures, Data Availability Section)
If your work benefited from core facilities, was their service mentioned in the acknowledgments section?	Not Applicable	

Design

Study protocol	Information included in the manuscript?	In which section is the information available? (Reagents and Tools Table, Materials and Methods, Figures, Data Availability Section)
If study protocol has been **pre-registered, provide DOI in the manuscript**. For clinical trials, provide the trial registration number **OR** cite DOI.	Not Applicable	
Report the **clinical trial registration number** (at ClinicalTrials.gov or equivalent), where applicable.	Not Applicable	

Laboratory protocol	Information included in the manuscript?	In which section is the information available? (Reagents and Tools Table, Materials and Methods, Figures, Data Availability Section)
Provide DOI OR other citation details if **external detailed step-by-step protocols** are available.	Not Applicable	

Experimental study design and statistics	Information included in the manuscript?	In which section is the information available? (Reagents and Tools Table, Materials and Methods, Figures, Data Availability Section)
Include a statement about **sample size** estimate even if no statistical methods were used.	Yes	Appendix Table S4
Were any steps taken to minimize the effects of subjective bias when allocating animals/samples to treatment (e.g. **randomization procedure**)? If yes, have they been described?	Not Applicable	
Include a statement about **blinding** even if no blinding was done.	Not Applicable	
Describe **inclusion/exclusion criteria** if samples or animals were excluded from the analysis. Were the criteria pre-established? If sample or data points were omitted from analysis, report if this was due to attrition or intentional exclusion and provide justification.	Not Applicable	
For every figure, are **statistical tests** justified as appropriate? Do the data meet the assumptions of the tests (e.g., normal distribution)? Describe any methods used to assess it. Is there an estimate of variation within each group of data? Is the variance similar between the groups that are being statistically compared?	Yes	Materials and Methods

Sample definition and in-laboratory replication	Information included in the manuscript?	In which section is the information available? (Reagents and Tools Table, Materials and Methods, Figures, Data Availability Section)
In the figure legends: state number of times the experiment was **replicated** in laboratory.	Yes	Figures
In the figure legends: define whether data describe **technical or biological replicates**.	Yes	Figures, Materials and Methods

Ethics

Ethics	Information included in the manuscript?	In which section is the information available? (Reagents and Tools Table, Materials and Methods, Figures, Data Availability Section)
Studies involving **human participants**: State details of **authority granting ethics approval** (IRB or equivalent committee(s), provide reference number for approval.	Not Applicable	
Studies involving **human participants**: Include a statement confirming that **informed consent** was obtained from all subjects and that the experiments conformed to the principles set out in the WMA Declaration of Helsinki and the Department of Health and Human Services Belmont Report.	Not Applicable	
Studies involving **human participants:** For publication of **patient photos**, include a statement confirming that consent to publish was obtained.	Not Applicable	
Studies involving experimental **animals**: State details of **authority granting ethics approva**l (IRB or equivalent committee(s), provide reference number for approval. Include a statement of compliance with ethical regulations.	Not Applicable	
Studies involving **specimen and field samples:** State if relevant **permits** obtained, provide details of authority approving study; if none were required, explain why.	Not Applicable	

Dual Use Research of Concern (DURC)	Information included in the manuscript?	In which section is the information available? (Reagents and Tools Table, Materials and Methods, Figures, Data Availability Section)
Could your study fall under dual use research restrictions? Please check biosecurity documents and list of **select agents and toxins** (CDC): https://www.selectagents.gov/sat/list.htm	Not Applicable	
If you used a select agent, is the security level of the lab appropriate and reported in the manuscript?	Not Applicable	
If a study is subject to dual use research of concern regulations, is the name of the **authority granting approval and reference number** for the regulatory approval provided in the manuscript?	Not Applicable	

Reporting

The MDAR framework recommends adoption of discipline-specific guidelines, established and endorsed through community initiatives. Journals have their own policy about requiring specific guidelines and recommendations to complement MDAR.

Adherence to community standards	Information included in the manuscript?	In which section is the information available? (Reagents and Tools Table, Materials and Methods, Figures, Data Availability Section)
State if relevant guidelines or checklists (e.g., **ICMJE, MIBBI, ARRIVE, PRISMA**) have been followed or provided.	Not Applicable	
For **tumor marker prognostic studies,** we recommend that you follow the **REMARK** reporting guidelines (see link list at top right). See author guidelines, under 'Reporting Guidelines'. Please confirm you have followed these guidelines.	Not Applicable	
For **phase II and III randomized controlled trials**, please refer to the **CONSORT** flow diagram (see link list at top right) and submit the CONSORT checklist (see link list at top right) with your submission. See author guidelines, under 'Reporting Guidelines'. Please confirm you have submitted this list.	Not Applicable	

Data Availability

Data availability	Information included in the manuscript?	In which section is the information available? (Reagents and Tools Table, Materials and Methods, Figures, Data Availability Section)
Have **primary datasets** been deposited according to the journal's guidelines (see 'Data Deposition' section) and the respective accession numbers provided in the Data Availability Section?	Yes	Data Availability section
Were **human clinical and genomic datasets** deposited in a public access-controlled repository in accordance to ethical obligations to the patients and to the applicable consent agreement?	Not Applicable	
Are **computational models** that are central and integral to a study available without restrictions in a machine-readable form? Were the relevant accession numbers or links provided?	Yes	Materials and Methods, Data Availability Section
If publicly available data were reused, provide the respective **data citations in the reference list**.	Not Applicable	
